# Supplementary material for: A core phyllosphere microbiome exists across distant populations of a tree species indigenous to New Zealand
Source: PLoS One. 2020 Aug 13;15(8):e0237079. doi: 10.1371/journal.pone.0237079 (PMC7425925; doi:10.1371/journal.pone.0237079)
Supplement: S1 Fig — Borders represent regional boundaries. Total rain (mm) and total sun (mm) data retrieved from the National Climate Database (NIWA) for the month prior to sampling. Map made with Natural Earth. (PDF) [file pone.0237079.s001.pdf]

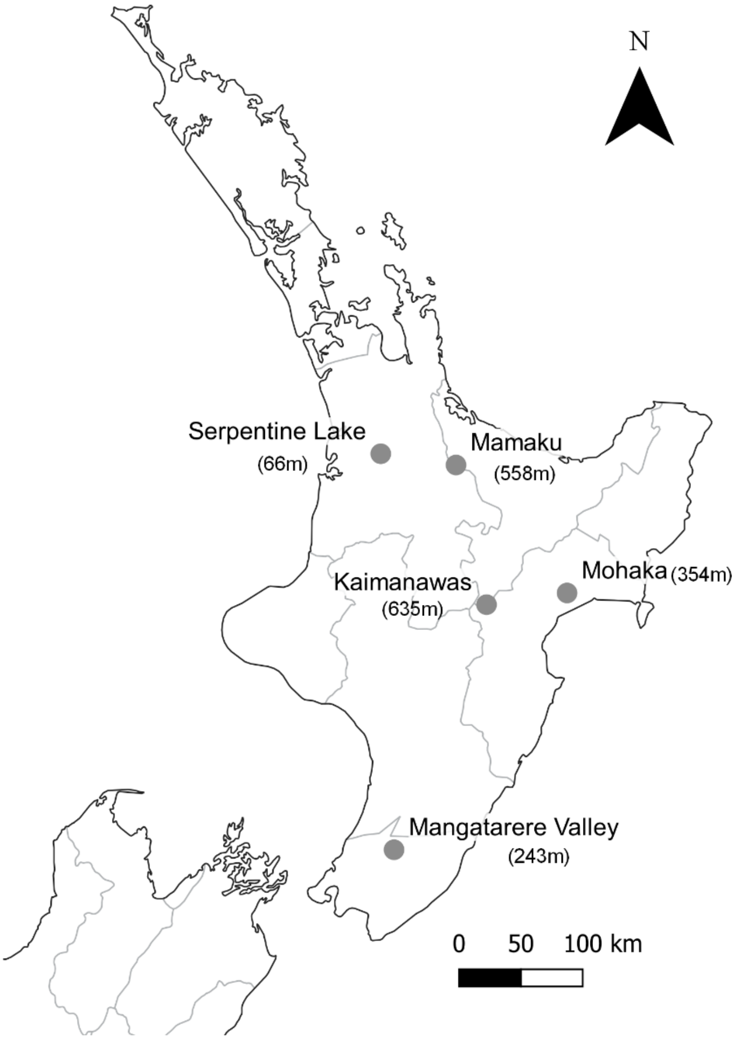

Site	Total rain (mm)	Total sun (hrs)
Mohaka	50	130
Serpentine Lake	112	87
Mangatarere Valley	91	105
Mamaku	65	97
Kaimanawa	56	110

S1 Fig: Location of *Leptospermum scoparium* (mānuka) populations in the North Island of New Zealand. Borders represent regional boundaries. Total rain (mm) and total sun (mm) data retrieved from the National Climate Database (NIWA) for the month prior to sampling. Map made with Natural Earth.
