# Supplementary material for: A core phyllosphere microbiome exists across distant populations of a tree species indigenous to New Zealand
Source: PLoS One. 2020 Aug 13;15(8):e0237079. doi: 10.1371/journal.pone.0237079 (PMC7425925; doi:10.1371/journal.pone.0237079)
Supplement: S2 Fig — Air temperature, relative humidity, and photosynthetically active radiation was measured at 15-minute intervals with a datalogger across the 24 h period prior to sampling. Each site is represented by colour. (PDF) [file pone.0237079.s002.pdf]

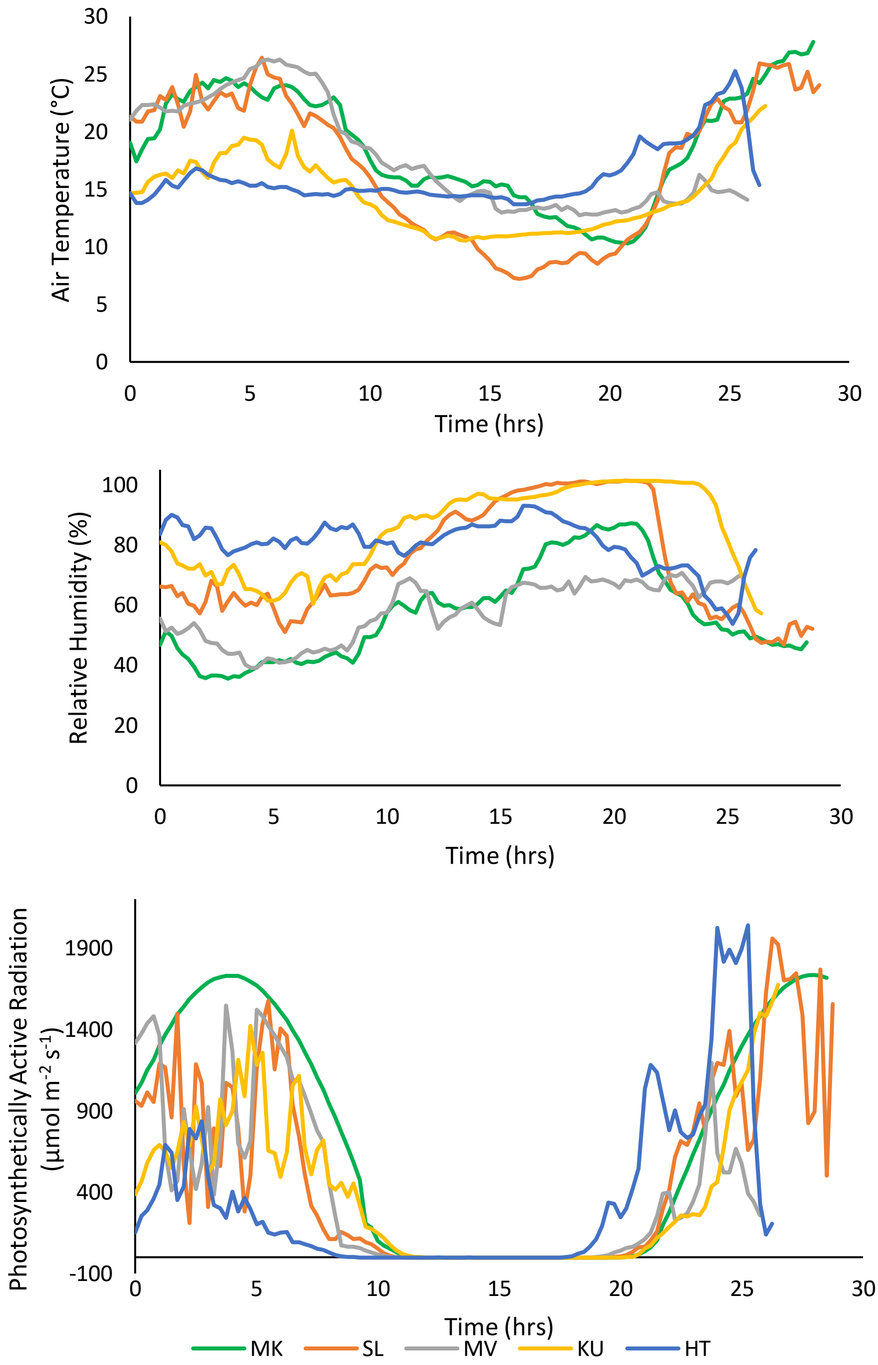

S2 Fig: Environmental conditions prior to sampling. Air temperature, relative humidity, and photosynthetically active radiation was measured at 15-minute intervals with a datalogger across the 24 h period prior to sampling. Each site is represented by colour.
