# Supplementary material for: A core phyllosphere microbiome exists across distant populations of a tree species indigenous to New Zealand
Source: PLoS One. 2020 Aug 13;15(8):e0237079. doi: 10.1371/journal.pone.0237079 (PMC7425925; doi:10.1371/journal.pone.0237079)
Supplement: S3 Fig — (a) Observed richness (ANOVA, p = 2.99 x 10^–6^, F = 9.26) was significantly higher at KU and MV compared to other sites. (b) Chao1 (ANOVA, 0.0023, F = 4.53) was significantly higher at KU compared to sites HT, MK, and MV. (PDF) [file pone.0237079.s003.pdf]

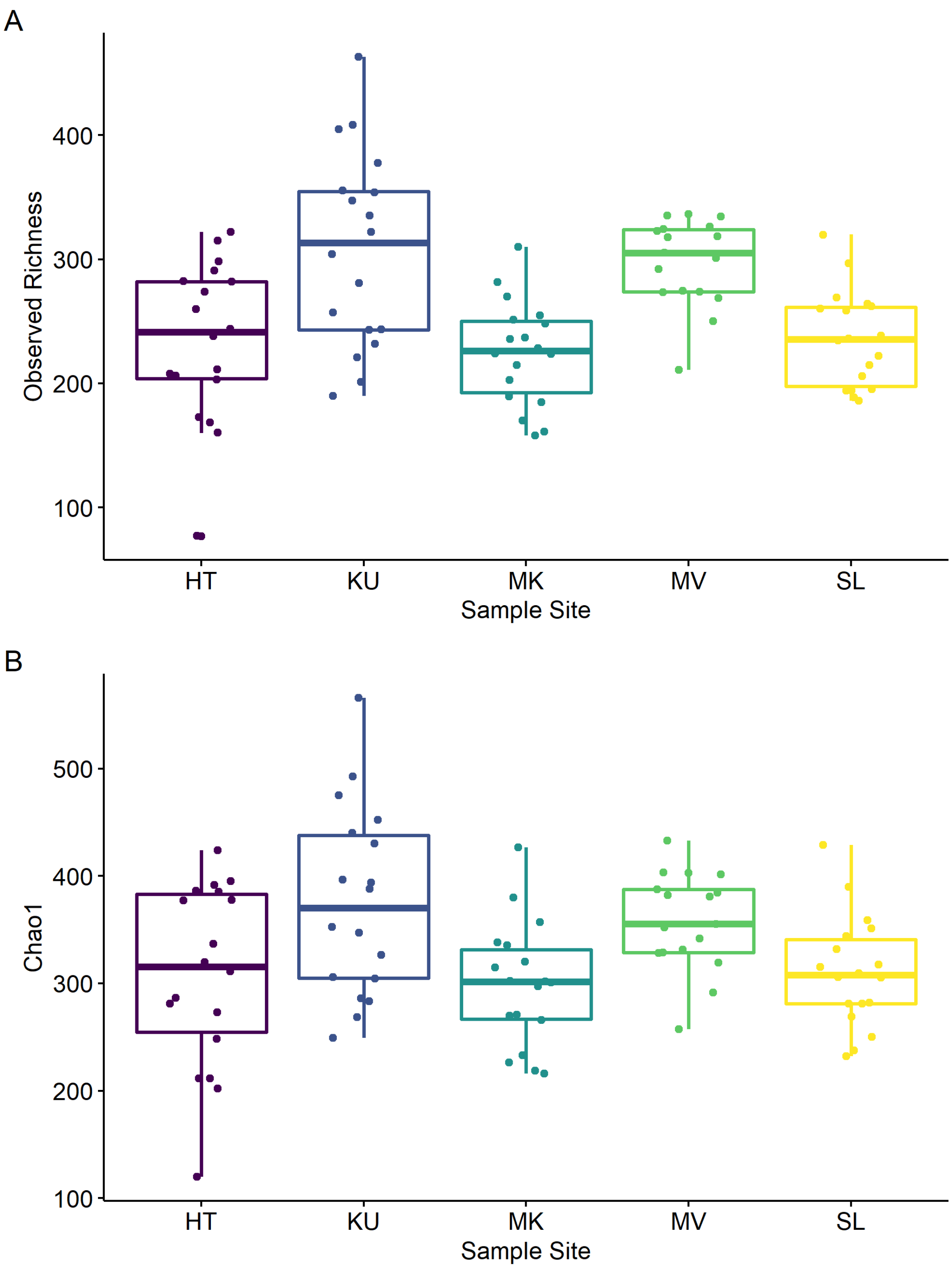

S3 Fig: Alpha diversity of phyllosphere communities from different sample sites. (a) Observed richness (ANOVA, p=2.99 x 10^{-6}, F = 9.26) was significantly higher at KU and MV compared to other sites. (b) Chao1 (ANOVA, 0.0023, F = 4.53) was significantly higher at KU compared to sites HT, MK, and MV.
