# Supplementary material for: A core phyllosphere microbiome exists across distant populations of a tree species indigenous to New Zealand
Source: PLoS One. 2020 Aug 13;15(8):e0237079. doi: 10.1371/journal.pone.0237079 (PMC7425925; doi:10.1371/journal.pone.0237079)
Supplement: S4 Fig — Bars represent the average relative abundance per sample tree (n = 3) and are grouped by site (HT, KU, MK, MV, SL). Phyla are represented by colour. (PDF) [file pone.0237079.s004.pdf]

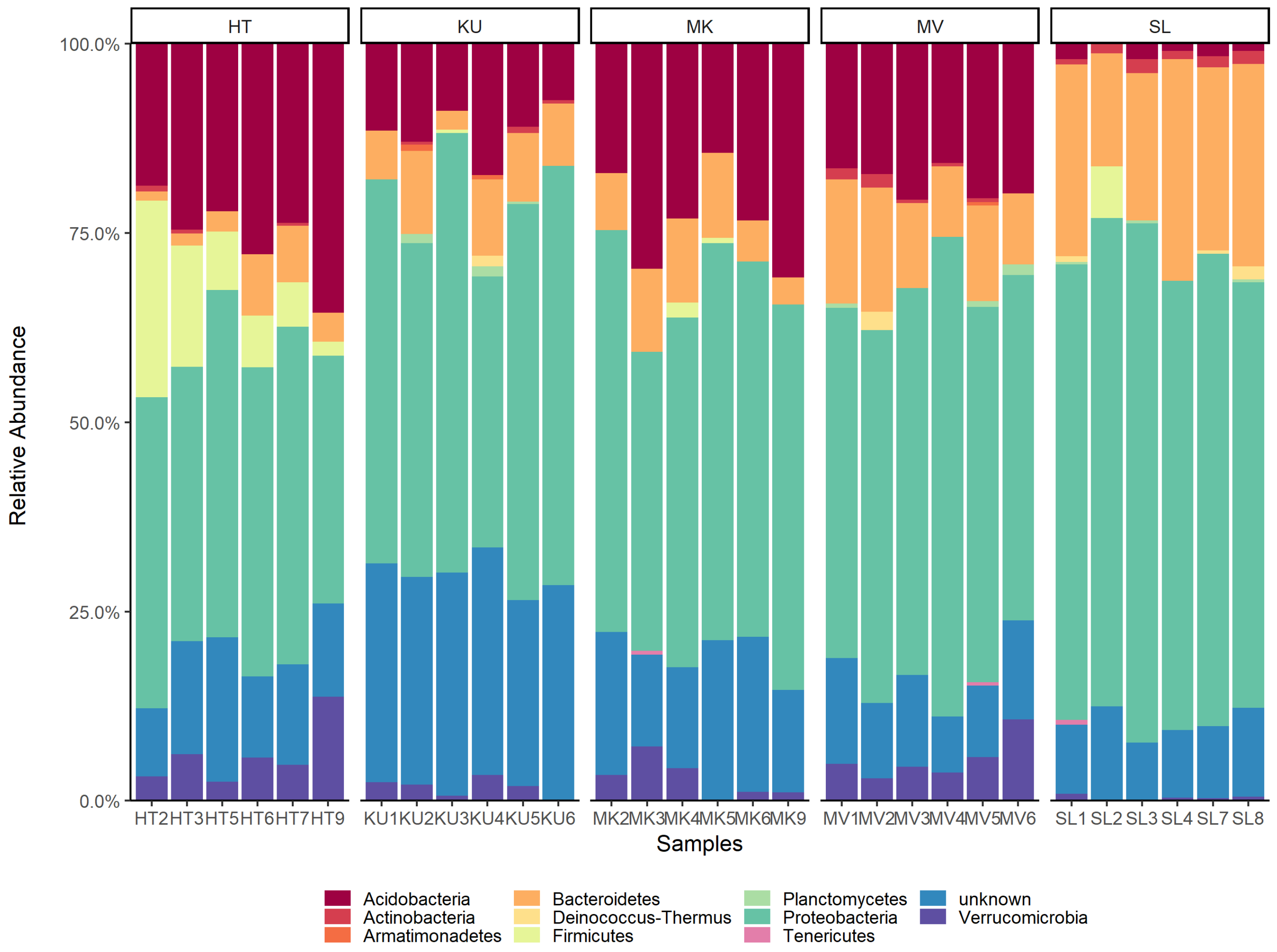

S4 Fig: Average relative abundance of phyla in the phyllosphere microbiome. Bars represent the average relative abundance per sample tree (n=3) and are grouped by site (HT, KU, MK, MV, SL). Phyla are represented by colour.
