# Supplementary material for: A core phyllosphere microbiome exists across distant populations of a tree species indigenous to New Zealand
Source: PLoS One. 2020 Aug 13;15(8):e0237079. doi: 10.1371/journal.pone.0237079 (PMC7425925; doi:10.1371/journal.pone.0237079)
Supplement: S5 Fig — Bars represent one sample. Samples are grouped by site (HT, KU, MK, MV, SL). Phyla are represented by colour. (PDF) [file pone.0237079.s005.pdf]

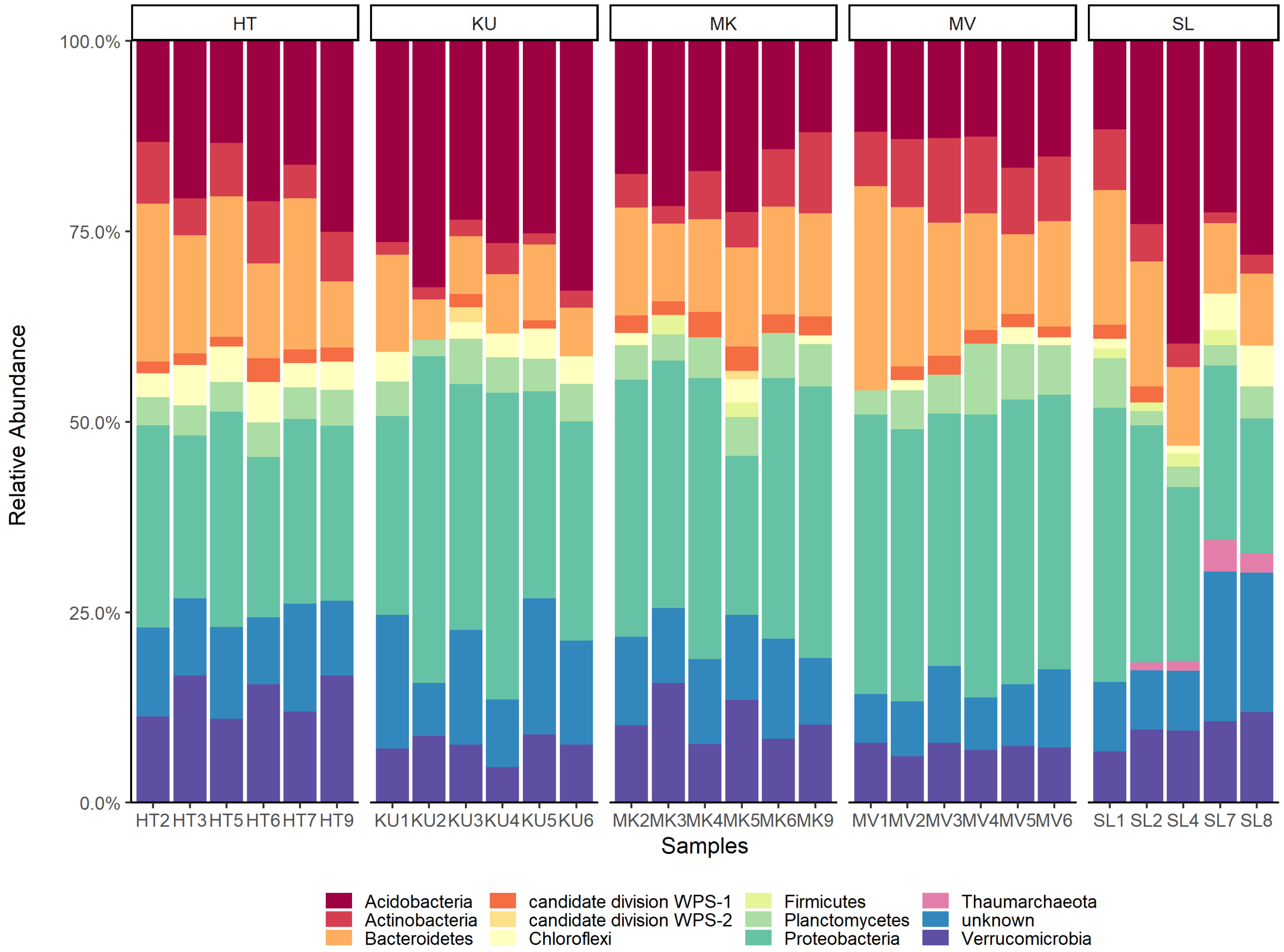

S5 Fig: Relative abundance of phyla in the soil microbiome surrounding mānuka trees. Bars represent one sample. Samples are grouped by site (HT, KU, MK, MV, SL). Phyla are represented by colour.
