# Supplementary material for: A core phyllosphere microbiome exists across distant populations of a tree species indigenous to New Zealand
Source: PLoS One. 2020 Aug 13;15(8):e0237079. doi: 10.1371/journal.pone.0237079 (PMC7425925; doi:10.1371/journal.pone.0237079)
Supplement: S6 Fig — Mānuka phyllosphere community dissimilarity within (A) and between (B) trees at each sample site. Community dissimilarity is based on Bray Curtis dissimilarity values. Boxes represent the interquartile range dissimilarity values. The thick bars represent the median of dissimilarity values, and the vertical segments extend to the fifth and the 95th percentiles of the distribution of values. Wilcoxon test statistically significant differences are shown with asterisks (*p<0.05, **p<0.01, ***p<0.001, ****p<0.0001). (PDF) [file pone.0237079.s006.pdf]

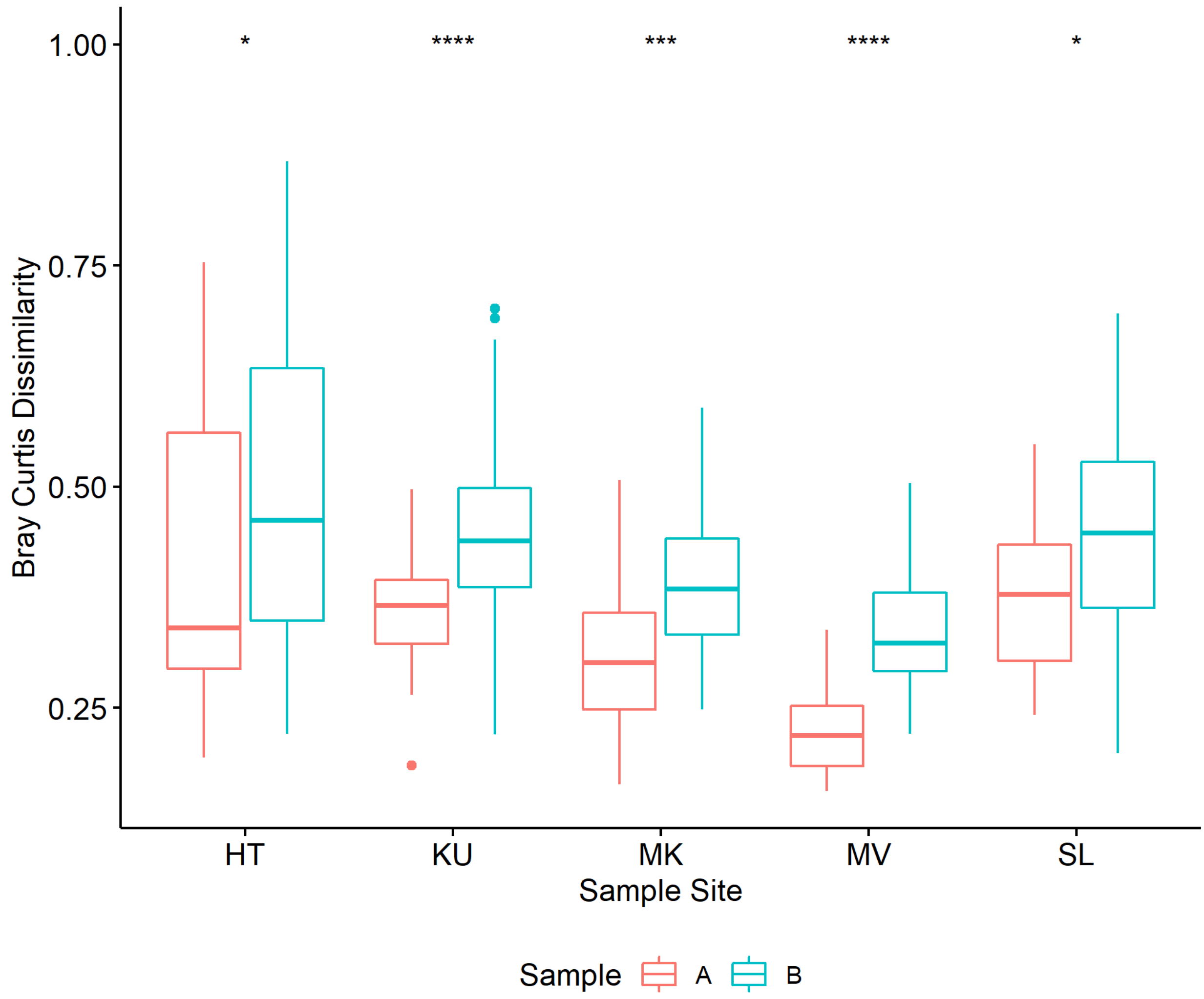

S6 Fig: Mānuka phyllosphere community dissimilarity within (A) and between (B) trees at each sample site. Community dissimilarity is based on Bray Curtis dissimilarity values. Boxes represent the interquartile range dissimilarity values. The thick bars represent the median of dissimilarity values, and the vertical segments extend to the fifth and the 95th percentiles of the distribution of values. Wilcoxon test statistically significant differences are shown with asterisks (*p<0.05, **p<0.01, ***p<0.001, ****p<0.0001).
