# Supplementary material for: A core phyllosphere microbiome exists across distant populations of a tree species indigenous to New Zealand
Source: PLoS One. 2020 Aug 13;15(8):e0237079. doi: 10.1371/journal.pone.0237079 (PMC7425925; doi:10.1371/journal.pone.0237079)
Supplement: S7 Fig — Branches are coloured according to sample region and sample date. Boxes indicate five cluster groups (k). Branch labels correspond to individual treeID. The length of the branches corresponds with Bray-Curtis dissimilarity between samples. (PDF) [file pone.0237079.s007.pdf]

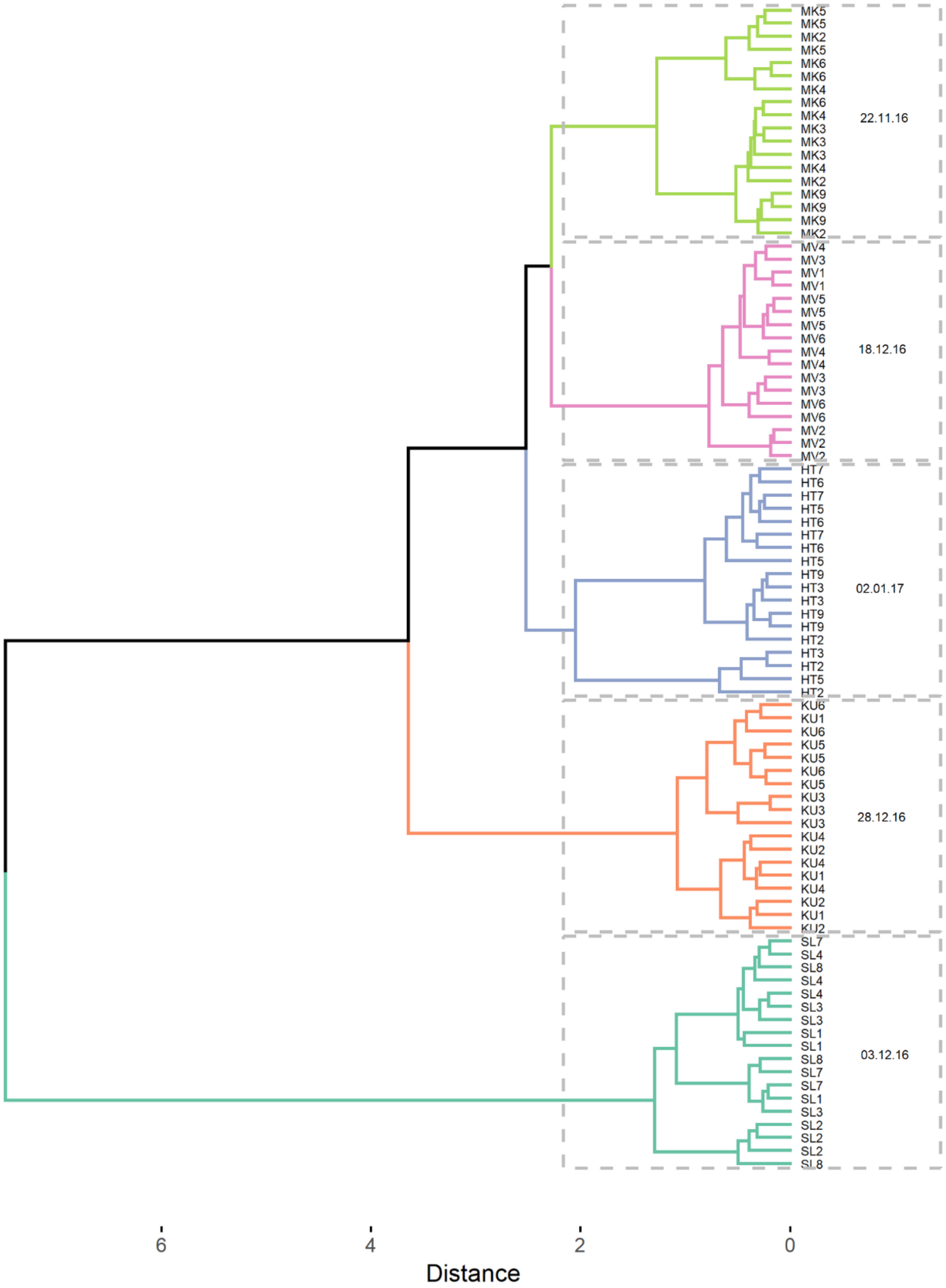

S7 Fig: Hierarchical clustering analysis of the mānuka phyllosphere microbiome. Branches are coloured according to sample sites and sample dates. Boxes indicate five cluster groups (k). Branch labels correspond to individual treeID. The length of the branches corresponds with Bray-Curtis dissimilarity between samples.
