# Supplementary material for: A core phyllosphere microbiome exists across distant populations of a tree species indigenous to New Zealand
Source: PLoS One. 2020 Aug 13;15(8):e0237079. doi: 10.1371/journal.pone.0237079 (PMC7425925; doi:10.1371/journal.pone.0237079)
Supplement: S8 Fig — Community dissimilarity (y axis) is based on Bray Curtis. Environmental dissimilarity (x axis) is based on Euclidean distances of average night temperature (A), day-night temperature differential (B), monthly rain (C), monthly cloud cover (D), and monthly sun hours (E). Line represents Pearson product moment correlation coefficient (R). (PDF) [file pone.0237079.s008.pdf]

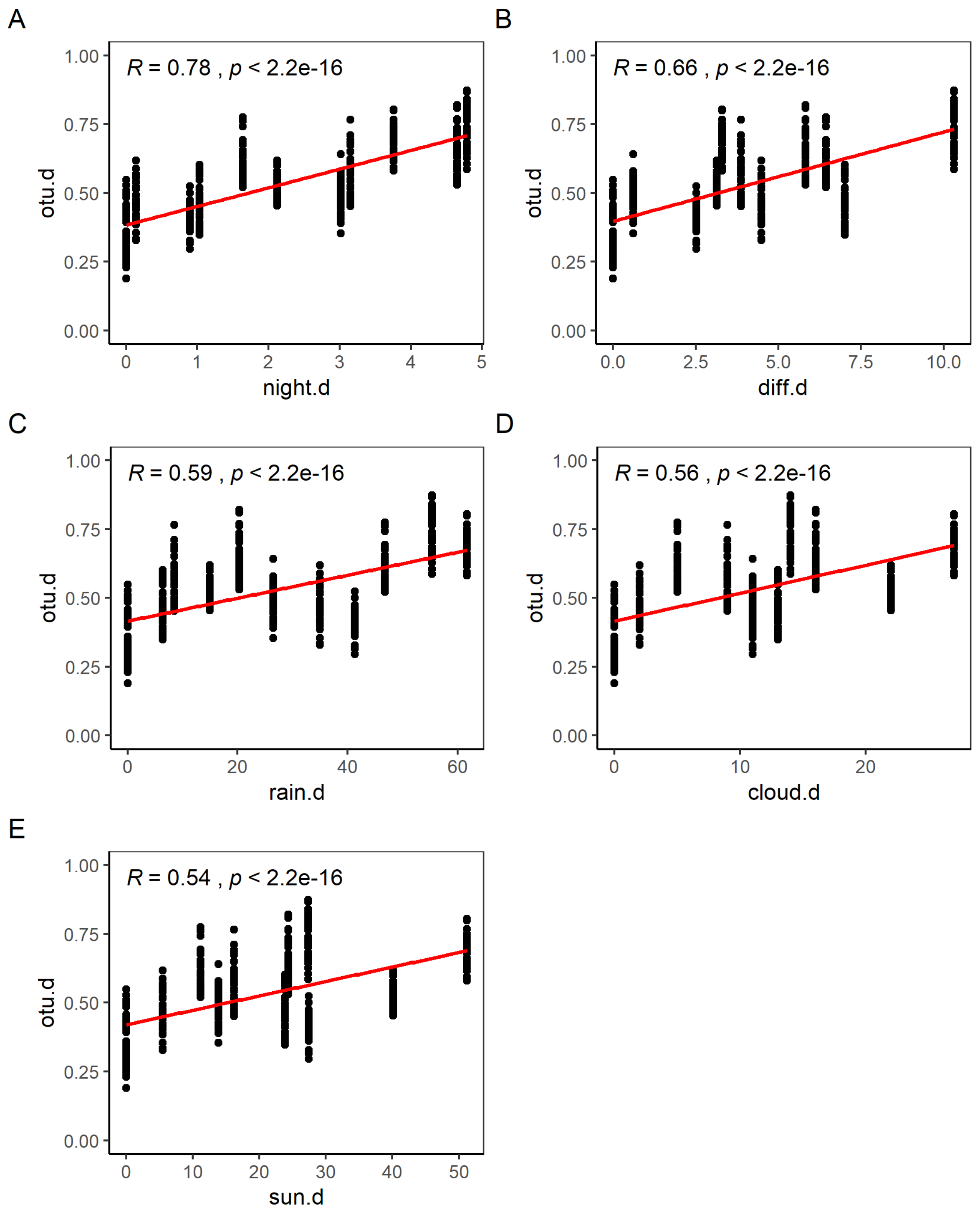

S8 Fig: Correlation between mānuka phyllosphere total community dissimilarity and environmental dissimilarity. Community dissimilarity (y axis) is based on Bray Curtis. Environmental dissimilarity (x axis) is based on Euclidean distances of average night temperature (A), day-night temperature differential (B), monthly rain (C), monthly cloud cover (D), and monthly sun hours (E). Line represents Pearson product moment correlation coefficient (*R*).
