# Supplementary material for: A core phyllosphere microbiome exists across distant populations of a tree species indigenous to New Zealand
Source: PLoS One. 2020 Aug 13;15(8):e0237079. doi: 10.1371/journal.pone.0237079 (PMC7425925; doi:10.1371/journal.pone.0237079)
Supplement: S9 Fig — Community dissimilarity (y axis) of non-core (left panels) and core (right panels) taxa is based on Bray Curtis. Environmental dissimilarity (x axis) is based on Euclidean distances of average night temperature (AB), day-night temperature differential (CD), monthly rain (EF), monthly cloud cover (GH), and monthly sun hours (IJ). Line represents Pearson product moment correlation coefficient (R). (PDF) [file pone.0237079.s009.pdf]

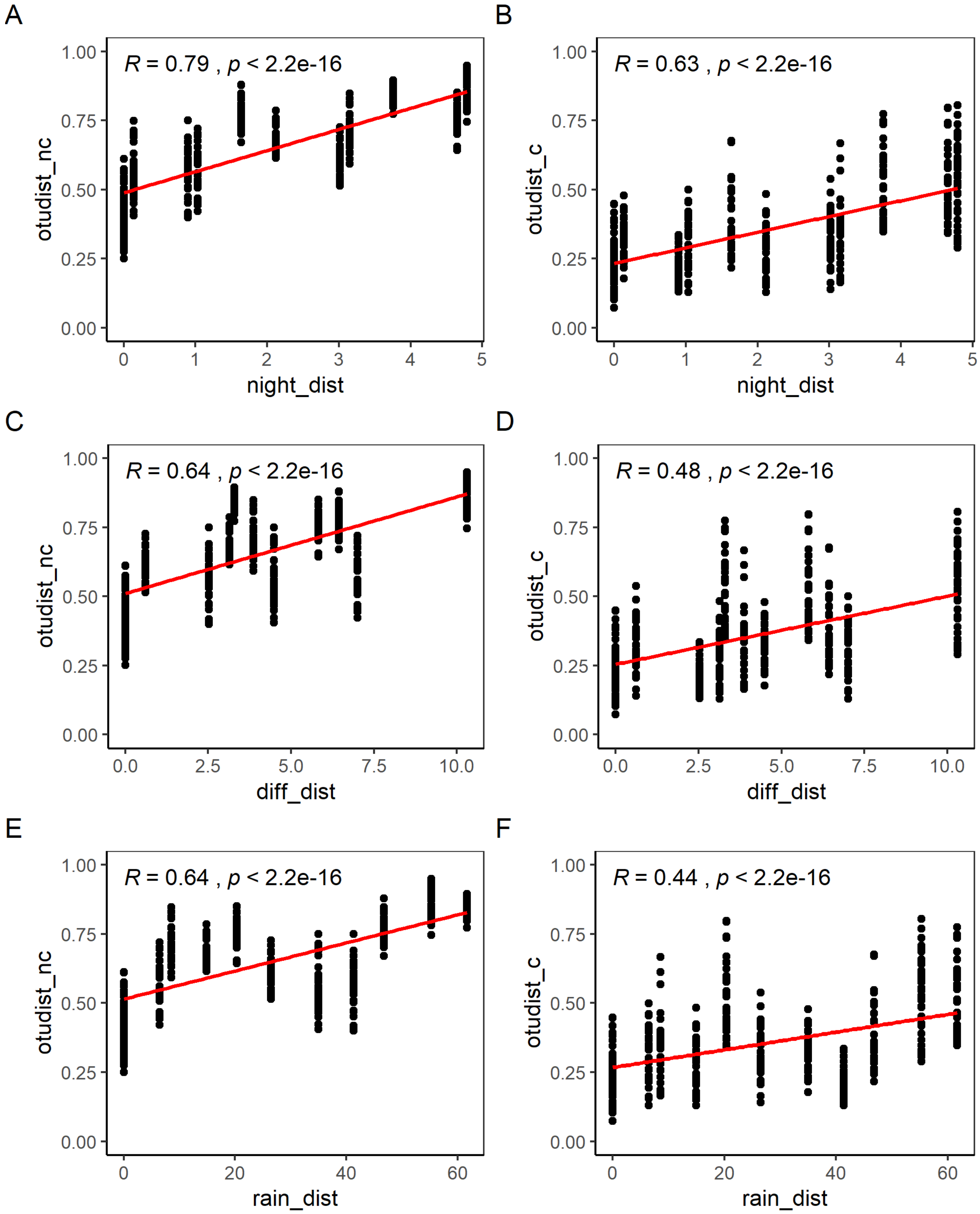

A
R = 0.79 , p < 2.2e-16
otudist_nc
night_dist
B
R = 0.63 , p < 2.2e-16
otudist_c
night_dist
C
R = 0.64 , p < 2.2e-16
otudist_nc
diff_dist
D
R = 0.48 , p < 2.2e-16
otudist_c
diff_dist
E
R = 0.64 , p < 2.2e-16
otudist_nc
rain_dist
F
R = 0.44 , p < 2.2e-16
otudist_c
rain_dist

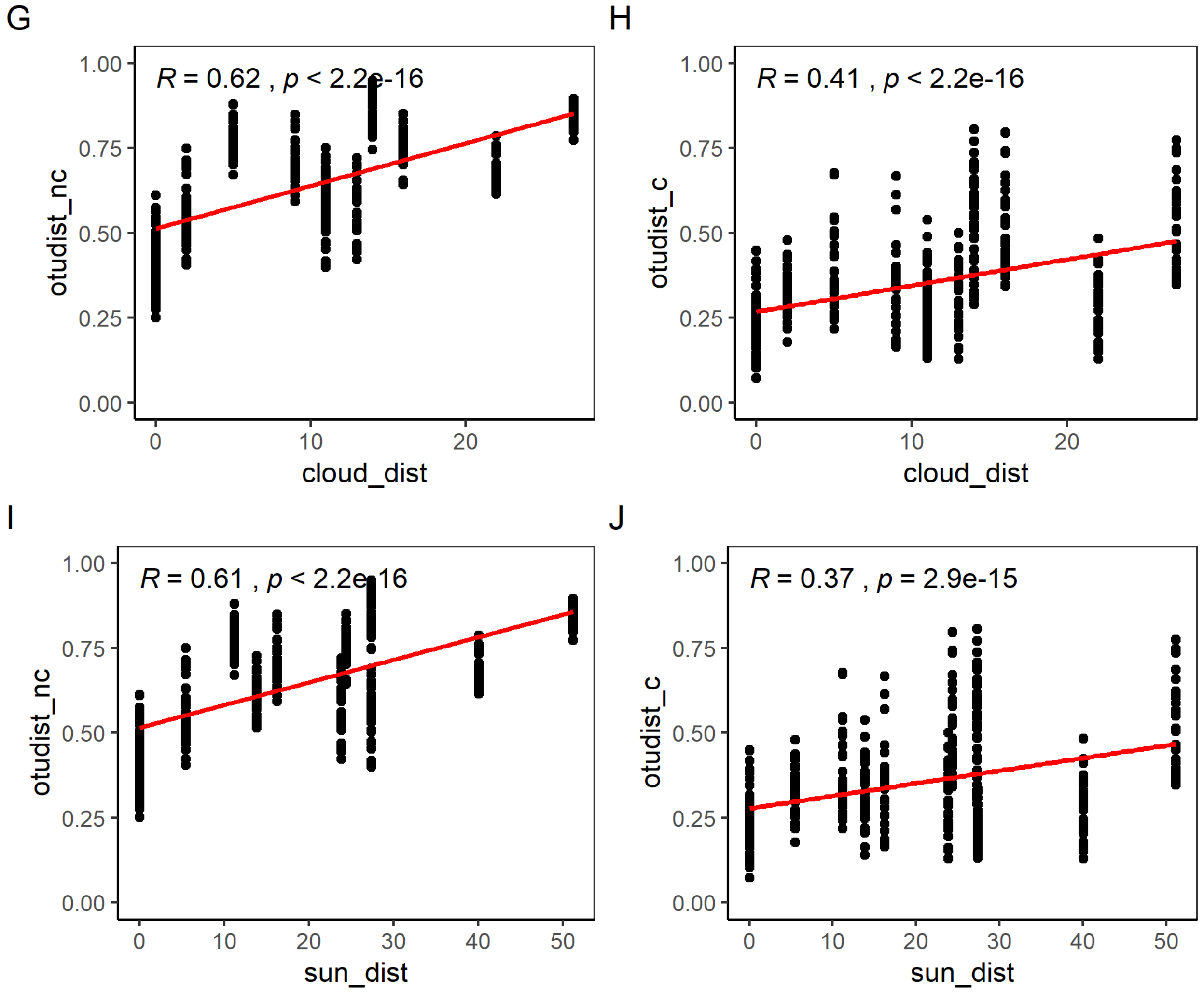

S9 Fig: Correlation between mānuka phyllosphere community dissimilarity and environmental dissimilarity for core and non-core taxa. Community dissimilarity (y axis) of non-core (left panels) and core (right panels) taxa is based on Bray Curtis. Environmental dissimilarity (x axis) is based on Euclidean distances of average night temperature (AB), day-night temperature differential (CD), monthly rain (EF), monthly cloud cover (GH), and monthly sun hours (IJ). Line represents Pearson product moment correlation coefficient (R).
