# Supplementary material for: A core phyllosphere microbiome exists across distant populations of a tree species indigenous to New Zealand
Source: PLoS One. 2020 Aug 13;15(8):e0237079. doi: 10.1371/journal.pone.0237079 (PMC7425925; doi:10.1371/journal.pone.0237079)
Supplement: S10 Fig — Isolates were identified using BLASTn; the accession number and isolation source of each isolate is provided. Sequences were aligned with ClustalW. Bootstrap probabilities based on 1000 replications are shown. Bar: 0.05 nucleotide substitutions rate (Knuc) units. (PDF) [file pone.0237079.s010.pdf]

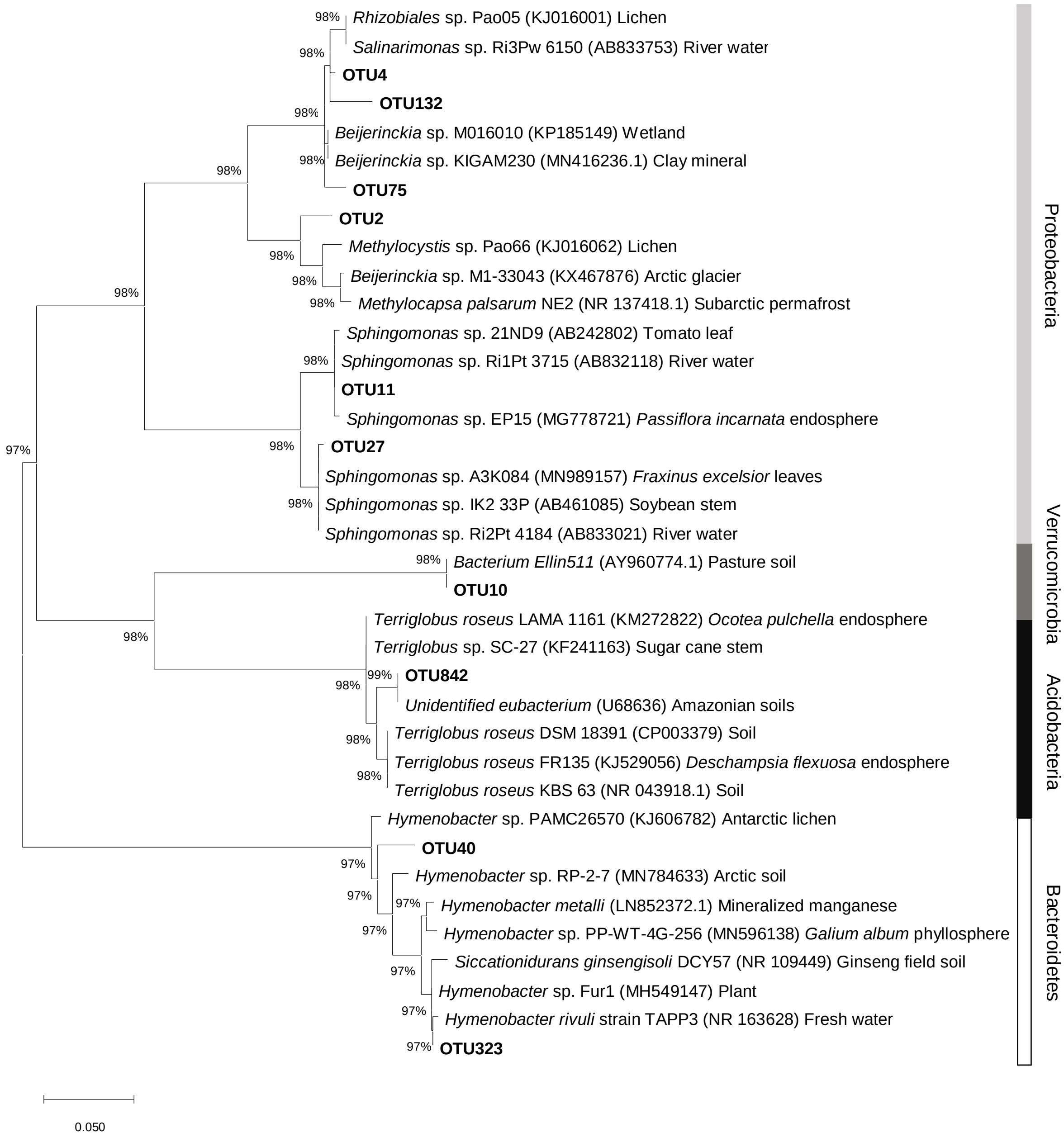

S10 Fig: Phylogenetic maximum-likelihood analysis based on 16S rRNA gene PCR amplicon sequences of core phyllosphere OTUs and bacterial isolates with the closest sequence identity. Isolates were identified using BLASTn; the accession number and isolation source of each isolate is provided. Sequences were aligned with ClustalW. Bootstrap probabilities based on 1000 replications are shown. Bar: 0.05 nucleotide substitutions rate (Knuc) units.
