# Supplementary material for: A core phyllosphere microbiome exists across distant populations of a tree species indigenous to New Zealand
Source: PLoS One. 2020 Aug 13;15(8):e0237079. doi: 10.1371/journal.pone.0237079 (PMC7425925; doi:10.1371/journal.pone.0237079)
Supplement: S11 Fig — Estimates of log2 fold changes were obtained using DESeq2. Colour depicts members of the core phyllosphere microbiome. A full list of OTUs with significant differential abundances, defined by a DeSeq2 log-fold difference with an adjusted p value of ≤ 0.01, is presented in S8 Table. (PDF) [file pone.0237079.s011.pdf]

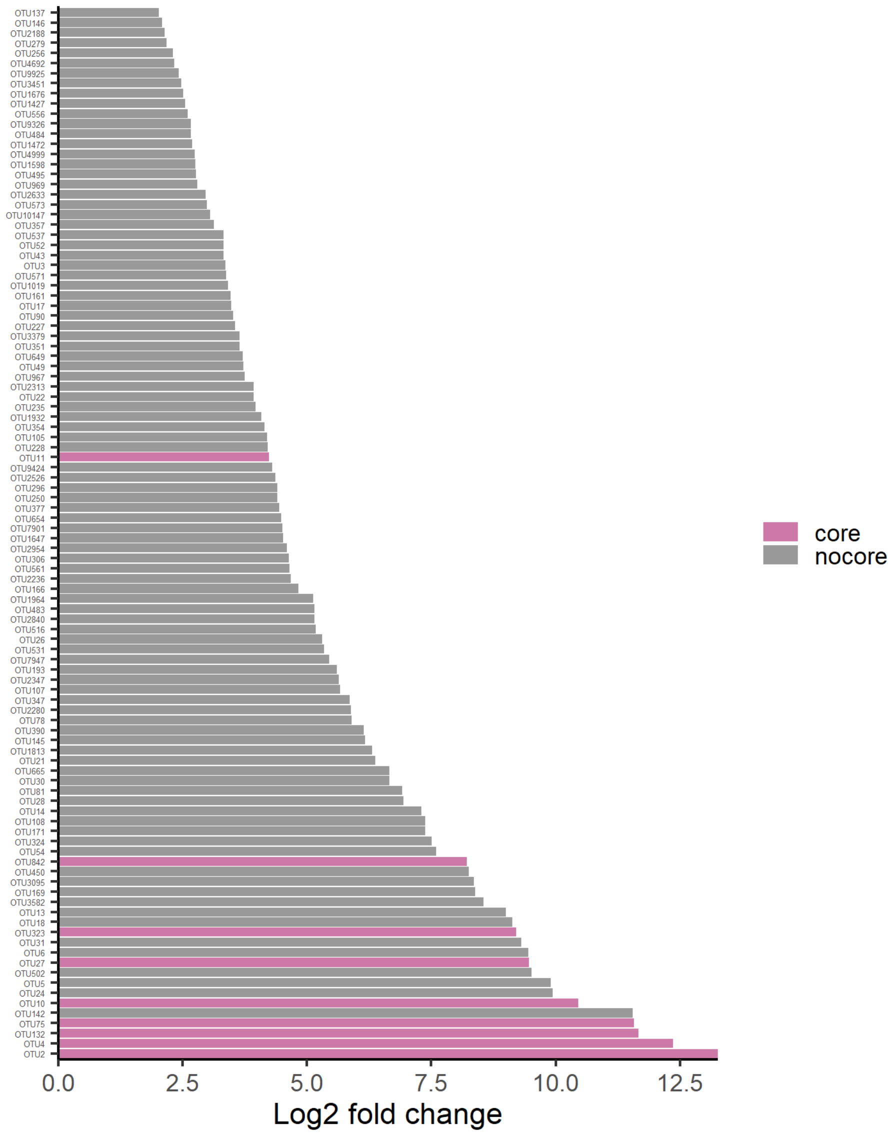

S11 Fig: OTUs with significantly increased abundance in the phyllosphere relative to soil (n=104). Estimates of log2 fold changes were obtained using DESeq2. Colour depicts members of the core phyllosphere microbiome. A full list of OTUs with significant differential abundances, defined by a DeSeq2 log-fold difference with an adjusted p value of $\leqslant 0.01$, is presented in S8 Table.
