# Supplementary material for: A core phyllosphere microbiome exists across distant populations of a tree species indigenous to New Zealand
Source: PLoS One. 2020 Aug 13;15(8):e0237079. doi: 10.1371/journal.pone.0237079 (PMC7425925; doi:10.1371/journal.pone.0237079)
Supplement: S1 Table — Monthly averages were obtained from the National Climate Database (NIWA). Other environmental conditions were measured at 15-minute intervals with a datalogger across the 24 h period prior to sampling. (PDF) [file pone.0237079.s012.pdf]

S1 Table: Spatial and environmental metadata for each mānuka sample site.

Site Information	MK	SL	MV	KU	HT
Sample date	2016-11-22	2016-12-03	2016-12-18	2016-12-28	2017-01-02
GPS - WGS 1984 (G1762) (degree minutes)	39°01 S 177°08 E	37°56 S 175°19 E	40°57 S 175°26 E	38°02 S 176°03 E	39°06 S 176°21 E
Average temperature (°C)	18.3	16.3	17.87932	14.5	15.5
Average day temperature (°C)	22.7	22.3	21.1	17.5	16.7
Average night temperature (°C)	13.5	9.7	14.4	11.3	14.5
Day-night temperature differential (°C)	9.3	12.6	6.7	6.1	2.3
Relative humidity day average (%)	43.7	60.5	52.2	72.5	78.6
Photosynthetically active radiation (µmol m^{-2} s^{-1})	1376	903.8	862.5	827.5	604.2
Monthly average temperature (°C)	19	17	16	18	18
Monthly total precipitation (mm)	50	112	91	65	56
Monthly average wind speed (mph)	10	20	7	9	10
Monthly average pressure (mb)	1007	1010	1008	1014	1014
Monthly average cloud cover (%)	31	58	42	53	44
Monthly average humidity (%)	67	83	79	77	76
Monthly total sun (hr)	130	87	105	97	110

Monthly averages were obtained from the National Climate Database (NIWA). Other environmental conditions were measured at 15-minute intervals with a datalogger across the 24 h period prior to sampling.
