# Supplementary material for: A core phyllosphere microbiome exists across distant populations of a tree species indigenous to New Zealand
Source: PLoS One. 2020 Aug 13;15(8):e0237079. doi: 10.1371/journal.pone.0237079 (PMC7425925; doi:10.1371/journal.pone.0237079)
Supplement: S2 Table — (PDF) [file pone.0237079.s013.pdf]

S2 Table: Spatial metadata for each mānuka phyllosphere sample.

Site	Sample ID	Branch height (m)	Tree diameter (cm)	Branch aspect (°)	Tree ID	Elevation (m)	Tree height (m)
MK	MK2.4_03	1.03	2.3	135	MK2	357	2.7
	MK2.5_34	1.83	2.3	145			
	MK2.6_08	1.55	2.3	0			
	MK3.4_04	2.04	6.6	71	MK3	354	2.7
	MK3.5_36	2.25	6.6	120			
	MK3.6_37	2.54	6.6	111			
	MK4.4_06	2.2	4.0	80	MK4	358	3.18
	MK4.5_40	2.8	4.0	0			
	MK4.6_44	1.8	4.0	45			
	MK5.4_07	1.62	1.5	69	MK5	356	1.82
	MK5.5_41	1.28	1.5	128			
	MK5.6_45	1.4	1.5	0			
	MK6.4_86	1.8	3.0	139	MK6	357	2.52
	MK6.5_46	2.0	3.0	359			
	MK6.6_49	2.2	3.0	0			
	MK9.4_09	2.25	3.55	55	MK9	343	2.86
	MK9.5_50	2.6	3.55	60			
	MK9.6_51	2.53	3.55	75			
SL	SL1.4_10	2.4	8.9	91	SL1	66	4
	SL1.5_55	1.8	8.9	306			
	SL1.6_53	1.75	8.9	339			
	SL2.4_11	2.35	8.5	110	SL2	66	3.9
	SL2.5_58	2.1	8.5	118			
	SL2.6_56	2.25	8.5	160			
	SL3.4_12	2.15	12.8	93	SL3	66	4.1
	SL3.5_60	2.0	12.8	98			
	SL3.6_57	2.05	12.8	123			
	SL4.4_14	1.4	4.4	130	SL4	66	3.3
	SL4.5_61	1.7	4.4	140			
	SL4.6_05	1.25	4.4	150			
	SL7.4_13	1.9	4.5	12	SL7	66	3.3
	SL7.5_66	2.0	4.5	155			
	SL7.6_81	2.2	4.5	173			
	SL8.4_85	1.7	3.7	313	SL8	66	2.2
	SL8.5_03	1.8	3.7	9			
	SL8.6_34	1.4	3.7	75			
MV	MV1.4_04	1.85	9.7	270	MV1	245	3.3
	MV1.6_37	1.95	9.7	235			
	MV2.4_06	2.3	3.9	85	MV2	237	2.65
	MV2.5_40	2.3	3.9	10			
	MV2.6_44	2.35	3.9	110			
	MV3.4_07	2.6	4.4	195	MV3	240	4
	MV3.5_41	2.8	4.4	195			
	MV3.6_45	3.3	4.4	195			
	MV4.4_08	2.7	7.6	130	MV4	247	3.4
	MV4.5_46	2.6	7.6	160			
	MV4.6_49	2.6	7.6	0			

	MV5.4_09	2.05	3.5	280	MV5	246	2.85
	MV5.5_50	2.1	3.5	290			
	MV5.6_51	2.35	3.5	300			
	MV6.4_10	2.45	4.1	0	MV6	242	2.9
	MV6.5_55	2.7	4.1	120			
	MV6.6_53	2.0	4.1	145			
KU	KU1.4_11	1.15	1.5	300	KU1	561	2.3
	KU1.5_58	0.8	1.5	300			
	KU1.6_56	1.5	1.5	310			
	KU2.4_12	1.7	3.6	260	KU2	562	3.8
	KU2.5_60	2.1	3.6	10			
	KU2.6_57	3.1	3.6	175			
	KU3.4_14	1.9	2.3	65	KU3	562	2.5
	KU3.5_61	2.1	2.3	210			
	KU3.6_59	2.0	2.3	150			
	KU4.4_15	2.1	7.35	330	KU4	555	4
	KU4.5_63	2.5	7.35	235			
	KU4.6_25	2.3	7.35	70			
	KU5.4_22	1.9	15.75	120	KU5	555	3.7
	KU5.5_64	2.1	15.75	160			
	KU5.6_16	2.4	15.75	210			
	KU6.4_68	1.55	2.4	280	KU6	555	2.5
	KU6.5_18	1.7	2.4	15			
	KU6.6_65	2.0	2.4	30			
HT	HT2.4_17	1.2	3.5	205	HT2	638	2.8
	HT2.5_88	1.8	3.5	25			
	HT26_86	1.2	3.5	200			
	HT3.4_71	1.3	6.3	225	HT3	633	2.7
	HT3.5_19	1.5	6.3	250			
	HT3.6_89	1.1	6.3	210			
	HT5.4_72	1.0	7.2	230	HT5	632	2.8
	HT5.5_20	0.9	7.2	145			
	HT5.6_90	1.6	7.2	10			
	HT6.4_78	1.1	8.8	45	HT6	632	2.6
	HT6.5_21	1.55	8.8	115			
	HT6.6_91	1.7	8.8	45			
	HT7.4_80	1.2	17.6	205	HT7	638	3.1
	HT7.5_05	1.0	17.6	80			
	HT7.6_13	1.5	17.6	75			
	HT9.4_23	1.1	24.75	340	HT9	635	3.3
	HT9.5_24	1.2	24.75	25			
	HT9.6_48	0.6	24.75	345			
