# Supplementary material for: A core phyllosphere microbiome exists across distant populations of a tree species indigenous to New Zealand
Source: PLoS One. 2020 Aug 13;15(8):e0237079. doi: 10.1371/journal.pone.0237079 (PMC7425925; doi:10.1371/journal.pone.0237079)
Supplement: S3 Table — (PDF) [file pone.0237079.s014.pdf]

S3 Table: Number of samples, reads, and OTUs for total community, phyllosphere samples, and soil samples.

Variable	Leaf	Soil	Total
Samples (n)	89	29	118
Reads	928,317	736,849	1,665,157
OTUs	1,384	6,905	7,689
Average reads	10,430 ± 5,165	25,409 ± 4,932	14,111 ± 8,227
Average OTUs	256 ± 63	1,820 ± 314	640 ± 692
