# Supplementary material for: A core phyllosphere microbiome exists across distant populations of a tree species indigenous to New Zealand
Source: PLoS One. 2020 Aug 13;15(8):e0237079. doi: 10.1371/journal.pone.0237079 (PMC7425925; doi:10.1371/journal.pone.0237079)
Supplement: S4 Table — (PDF) [file pone.0237079.s015.pdf]

S4 Table: Mānuka phyllosphere microbiome raw alpha diversity values for observed richness, Shannon, and Chao1 indices.

SampleID	Observed	Shannon	Chao1
HT2.4_17	169	2.423	212
HT2.5_88	160	2.88	202
HT26_86	77	3.22	120
HT3.4_71	203	2.85	248
HT3.5_19	238	3.75	337
HT3.6_89	206	3.29	287
HT5.4_72	208	3.00	281
HT5.5_20	260	3.77	386
HT5.6_90	173	3.34	212
HT6.4_78	322	3.97	395
HT6.5_21	298	3.87	385
HT6.6_91	211	3.78	273
HT7.4_80	315	4.22	424
HT7.5_05	244	3.74	311
HT7.6_13	274	3.66	320
HT9.4_23	291	3.55	392
HT9.5_24	282	3.60	378
HT9.6_48	282	3.63	377
KU1.4_11	243	3.56	326
KU1.5_58	243	3.34	283
KU1.6_56	355	4.05	397
KU2.4_12	463	4.23	566
KU2.5_60	408	4.15	492
KU2.6_57	304	3.92	352
KU3.4_14	221	3.37	269
KU3.5_61	201	3.10	286
KU3.6_59	232	3.34	304
KU4.4_15	405	4.31	475
KU4.5_63	354	3.75	430
KU4.6_25	378	3.43	452
KU5.4_22	335	3.73	388
KU5.5_64	347	3.92	440
KU5.6_16	281	3.56	347
KU6.4_68	322	3.77	394
KU6.5_18	257	3.11	306
KU6.6_65	190	3.64	249
MK2.4_03	237	3.90	320
MK2.5_34	170	3.10	233
MK2.6_08	236	3.61	302
MK3.4_04	282	3.85	338
MK3.5_36	251	3.97	380

MK3.6_37	228	3.59	297
MK4.4_06	270	3.84	335
MK4.5_40	310	4.16	427
MK4.6_44	185	3.53	266
MK5.4_07	161	3.15	216
MK5.5_41	224	3.35	301
MK5.6_45	203	3.55	270
MK6.4_86	248	3.67	302
MK6.5_46	158	3.45	219
MK6.6_49	189	3.62	227
MK9.4_09	224	3.37	315
MK9.5_50	215	3.49	271
MK9.6_51	255	3.51	357
MV1.4_04	335	3.84	433
MV1.6_37	324	3.91	403
MV2.4_06	273	3.54	329
MV2.5_40	275	3.67	331
MV2.6_44	292	3.72	328
MV3.4_07	318	3.81	382
MV3.5_41	326	3.69	381
MV3.6_45	319	3.75	384
MV4.4_08	211	3.02	257
MV4.5_46	250	2.97	291
MV4.6_49	269	3.49	319
MV5.4_09	301	3.57	355
MV5.5_50	323	3.60	387
MV5.6_51	334	3.91	401
MV6.4_10	336	3.85	403
MV6.5_55	274	3.40	341
MV6.6_53	305	4.02	352
SL1.4_10	238	3.55	305
SL1.5_55	195	3.23	250
SL1.6_53	320	3.91	429
SL2.4_11	206	3.31	281
SL2.5_58	259	3.72	344
SL2.6_56	215	3.36	282
SL3.4_12	264	3.78	359
SL3.5_60	262	3.57	351
SL3.6_57	194	3.02	269
SL4.4_14	236	3.56	281
SL4.5_61	269	3.55	332
SL4.6_05	222	3.68	318
SL7.4_13	189	3.20	238
SL7.5_66	194	3.63	232
SL7.6_81	297	3.71	390
SL8.4_85	186	2.68	309

SL8.5_03	235	3.60	306
SL8.6_34	260	3.70	315
