# Supplementary material for: A core phyllosphere microbiome exists across distant populations of a tree species indigenous to New Zealand
Source: PLoS One. 2020 Aug 13;15(8):e0237079. doi: 10.1371/journal.pone.0237079 (PMC7425925; doi:10.1371/journal.pone.0237079)
Supplement: S5 Table — Statistically significant differences are shown with asterisks (*p<0.05, **p<0.01, ***p<0.001, ****p<0.0001). (PDF) [file pone.0237079.s016.pdf]

S5 Table: Site-wise Tukey test for mānuka phyllosphere microbiome richness and Chao1.

Site	Richness	Chao - 1
KU - HT	0.001**	0.030*
MK - HT	0.986	0.994
MV - HT	0.008**	0.204
SL - HT	0.999	0.999
MK - KU	0.0001***	0.009**
MV - KU	0.984	0.937
SL - KU	0.001**	0.041*
MV - MK	0.001**	0.086
SL - MK	0.976	0.985
SL - MV	0.010**	0.250

Statistically significant differences are shown with asterisks (*p<0.05, **p<0.01, ***p<0.001, ****p<0.0001).
