# Supplementary material for: A core phyllosphere microbiome exists across distant populations of a tree species indigenous to New Zealand
Source: PLoS One. 2020 Aug 13;15(8):e0237079. doi: 10.1371/journal.pone.0237079 (PMC7425925; doi:10.1371/journal.pone.0237079)
Supplement: S6 Table — Correlations calculated using Pearson’s product-moment correlation coefficient test (Corr). p > 0.5 correlation values are not shown. (PDF) [file pone.0237079.s017.pdf]

S6 Table: Correlation with mānuka phyllosphere microbiome alpha diversity and environmental parameters.

	Richness		Chao-1		Shannon	
	Corr	*P*	*Corr*	*P*	*Corr*	*P*
Average temperature (°C)	-	0.09	-	0.11	-	0.94
Average day temperature (°C)	-	0.08	-	0.13	-	0.87
Average night temperature (°C)	-	0.93	-	0.75	-	0.90
Day-night temperature differential (°C)	-	0.22	-	0.36	-	0.85
Relative humidity day average	-	0.36	-	0.36	-	0.67
Photosynthetically active radiation (µmol m^{-2} s^{-1})	-	0.10	-	0.19	-	0.64
Monthly average temperature (°C)	-0.27	0.01	-	0.08	-	0.85
Monthly total precipitation (mm)	-	0.47	-	0.62	-	0.66
Monthly average wind speed (mph)	-	0.08	-	0.33	-	0.64
Monthly average pressure (mb)	-	0.11	-	0.14	-	0.99
Monthly average cloud cover (%)	-	0.13	-	0.17	-	0.79
Monthly average humidity (%)	-	0.09	-	0.19	-	0.67
Monthly total sun (hr)	-0.21	0.05	-	0.10	-	0.86
Elevation (m)	-	0.44	-	0.48	-	0.71
Tree height (m)	0.40	0.0001	0.39	0.0001	0.23	0.03
Tree diameter (cm)	-	0.07	0.26	0.02	-	0.16
Branch height (m)	0.23	0.03	-	0.14	-	0.28
Branch aspect	-	0.09	0.24	0.03	-	0.51

Correlations calculated using Pearson's product-moment correlation coefficient test (Corr). p > 0.5 correlation values are not shown.
