# Supplementary material for: A core phyllosphere microbiome exists across distant populations of a tree species indigenous to New Zealand
Source: PLoS One. 2020 Aug 13;15(8):e0237079. doi: 10.1371/journal.pone.0237079 (PMC7425925; doi:10.1371/journal.pone.0237079)
Supplement: S7 Table — Relative abundance of core taxa in phyllosphere samples is averaged per tree and site (mean ± SD). (PDF) [file pone.0237079.s018.pdf]

S7 Table: Relative abundance of core taxa across all mānuka phyllosphere (n=89) and soil (n=29) samples.

Site	Tree	Sample	ID	OTU2	OTU4	OTU27	OTU10	OTU11	OTU40	OTU75	OTU132	OTU323	OTU842
HT	HT2	Leaf	HT2.4_17	0.80	2.50	0.10	1.15	0.15	0.23	0.58	0.40	0.01	0.13
		Leaf	HT2.5_88	0.10	25.33	0.31	0.05	0.05	0.39	9.66	4.41	0.02	0.03
		Leaf	HT26_86	4.18	11.01	0.95	7.48	0.37	1.39	1.61	2.35	0.07	2.05
		Average leaf		1.69 ± 1.78	12.94 ± 9.42	0.45 ± 0.36	2.90 ± 3.28	0.19 ± 0.13	0.67 ± 0.51	3.95 ± 4.06	2.39 ± 1.64	0.03 ± 0.03	0.74 ± 0.93
		Soil	HT2S_60	0.00	0.00	0.00	0.00	0.05	0.00	0.00	0.00	0.00	0.00
	HT3	Leaf	HT3.4_71	1.01	5.76	0.07	1.05	0.34	0.14	1.47	0.39	0.03	0.12
		Leaf	HT3.5_19	8.46	9.84	0.37	7.08	1.36	0.52	4.36	0.96	0.12	0.65
		Leaf	HT3.6_89	11.14	15.20	0.28	6.55	0.23	0.70	5.12	1.86	0.13	0.68
		Average leaf		6.87 ± 4.29	10.27 ± 3.87	0.24 ± 0.12	4.90 ± 2.73	0.64 ± 0.51	0.45 ± 0.23	3.65 ± 1.57	1.07 ± 0.60	0.09 ± 0.04	0.49 ± 0.26
		Soil	HT3S_57	0.00	0.00	0.00	0.00	0.02	0.00	0.00	0.01	0.00	0.00
	HT5	Leaf	HT5.4_72	9.71	7.01	0.20	1.12	0.31	0.08	2.63	1.26	0.02	0.36
		Leaf	HT5.5_20	15.27	7.27	1.05	4.08	3.72	0.41	2.88	2.80	0.28	1.38
		Leaf	HT5.6_90	14.37	9.15	4.24	0.33	1.02	0.69	3.02	5.86	0.13	0.18
		Average leaf		13.12 ± 2.44	7.81 ± 0.95	1.83 ± 1.74	1.84 ± 1.61	1.68 ± 1.47	0.40 ± 0.25	2.84 ± 0.16	3.31 ± 1.91	0.14 ± 0.10	0.64 ± 0.53
		Soil	HT5S_14	0.00	0.00	0.00	0.00	0.09	0.00	0.00	0.00	0.00	0.00
	HT6	Leaf	HT6.4_78	10.73	6.63	0.38	3.72	1.80	0.55	2.92	3.03	0.23	0.64
		Leaf	HT6.5_21	14.08	6.52	0.27	2.95	1.06	0.36	2.31	1.27	0.11	0.40
		Leaf	HT6.6_91	6.42	6.80	0.22	4.14	2.37	0.99	2.38	1.77	0.16	0.84

		Average leaf		10.41 ± 3.14	6.65 ± 0.11	0.29 ± 0.07	3.60 ± 0.49	1.74 ± 0.54	0.64 ± 0.26	2.54 ± 0.27	2.03 ± 0.74	0.17 ± 0.05	0.63 ± 0.18
		Soil	HT6S_61	0.00	0.00	0.00	0.01	0.05	0.00	0.00	0.00	0.00	0.01
	HT7	Leaf	HT7.4_80	13.31	8.96	0.91	2.42	2.50	0.59	3.04	0.97	0.87	0.96
		Leaf	HT7.5_05	9.96	7.96	0.65	4.02	1.87	0.42	3.58	4.37	0.15	0.54
		Leaf	HT7.6_13	11.53	9.20	0.62	3.55	6.25	0.40	3.26	3.26	0.19	0.37
		Average leaf		11.60 ± 1.37	8.71 ± 0.53	0.72 ± 0.13	3.33 ± 0.67	3.54 ± 1.93	0.47 ± 0.09	3.29 ± 0.22	2.87 ± 1.41	0.41 ± 0.33	0.62 ± 0.25
		Soil	HT7S_59	0.00	0.00	0.00	0.00	0.09	0.00	0.00	0.00	0.00	0.01
	HT9	Leaf	HT9.4_23	8.91	12.48	0.14	9.48	0.19	0.99	4.03	0.75	0.06	0.43
		Leaf	HT9.5_24	5.83	8.10	0.32	13.23	0.30	0.59	2.85	1.00	0.09	0.61
		Leaf	HT9.6_48	5.32	9.73	0.38	12.18	0.44	0.31	3.19	0.72	0.13	0.60
		Average leaf		6.69 ± 1.59	10.10 ± 1.81	0.28 ± 0.10	11.63 ± 1.58	0.31 ± 0.10	0.63 ± 0.28	3.36 ± 0.49	0.82 ± 0.12	0.09 ± 0.03	0.54 ± 0.08
		Soil	HT9S_15	0.00	0.01	0.00	0.00	0.02	0.00	0.00	0.00	0.00	0.01
KU	KU1	Leaf	KU1.4_11	7.23	7.89	0.67	0.79	13.51	0.65	2.85	5.64	0.62	1.08
		Leaf	KU1.5_58	11.97	14.60	0.96	0.37	4.37	0.40	6.07	7.27	1.10	1.29
		Leaf	KU1.6_56	6.23	9.17	0.47	2.35	7.07	0.59	2.76	2.08	1.48	1.74
		Average leaf		8.48 ± 2.51	10.55 ± 2.91	0.70 ± 0.20	1.17 ± 0.85	8.32 ± 3.84	0.55 ± 0.11	3.89 ± 1.54	5.00 ± 2.17	1.07 ± 0.35	1.37 ± 0.27
		Soil	KU1S_55	0.00	0.01	0.00	0.00	0.01	0.00	0.00	0.00	0.00	0.00
	KU2	Leaf	KU2.4_12	4.26	6.50	0.55	1.08	3.71	0.40	1.47	1.23	0.47	0.49
		Leaf	KU2.5_60	4.95	11.89	0.51	1.53	10.98	0.83	2.49	3.49	0.55	1.16
		Leaf	KU2.6_57	7.85	8.50	0.92	1.37	10.99	0.82	1.79	2.78	1.13	0.42

		Average leaf		5.69 ± 1.56	8.97 ± 2.22	0.66 ± 0.18	1.32 ± 0.19	8.56 ± 3.43	0.68 ± 0.20	1.92 ± 0.43	2.50 ± 0.94	0.72 ± 0.29	0.69 ± 0.34
		Soil	KU2S_53	0.00	0.00	0.00	0.00	0.09	0.00	0.00	0.00	0.00	0.00
	KU3	Leaf	KU3.4_14	22.83	4.97	0.44	1.95	5.11	0.76	2.18	6.64	0.53	0.11
		Leaf	KU3.5_61	11.62	2.32	0.35	0.34	6.81	0.99	0.57	16.54	0.52	0.11
		Leaf	KU3.6_59	22.66	2.72	0.16	0.76	1.89	0.63	1.11	5.86	0.49	0.07
		Average leaf		19.04 ± 5.25	3.34 ± 1.17	0.32 ± 0.12	1.01 ± 0.68	4.61 ± 2.04	0.79 ± 0.15	1.29 ± 0.67	9.68 ± 4.86	0.51 ± 0.02	0.10 ± 0.02
		Soil	KU3S_11	0.00	0.00	0.00	0.00	0.01	0.00	0.00	0.00	0.00	0.00
	KU4	Leaf	KU4.4_15	6.68	8.77	0.16	2.47	1.18	1.82	2.72	1.54	0.56	1.35
		Leaf	KU4.5_63	10.39	11.25	0.21	1.96	0.84	2.31	4.66	3.77	0.74	0.91
		Leaf	KU4.6_25	5.26	9.06	0.41	0.97	3.59	0.33	1.25	2.78	0.41	0.89
		Average leaf		7.44 ± 2.16	9.69 ± 1.11	0.26 ± 0.11	1.80 ± 0.62	1.87 ± 1.23	1.49 ± 0.84	2.87 ± 1.40	2.67 ± 0.91	0.57 ± 0.14	1.05 ± 0.21
		Soil	KU4S_58	0.00	0.00	0.00	0.00	0.05	0.00	0.00	0.00	0.00	0.00
	KU5	Leaf	KU5.4_22	14.87	12.80	1.26	0.67	2.26	1.38	1.83	6.01	0.26	0.99
		Leaf	KU5.5_64	14.41	10.26	0.99	1.32	1.93	2.46	3.44	2.36	0.24	0.99
		Leaf	KU5.6_16	18.03	7.37	1.11	0.76	3.28	1.99	2.21	4.50	0.40	0.99
		Average leaf		15.77 ± 1.61	10.14 ± 2.22	1.12 ± 0.11	0.92 ± 0.29	2.49 ± 0.58	1.94 ± 0.44	2.49 ± 0.69	4.29 ± 1.49	0.30 ± 0.07	0.99 ± 0.00
		Soil	KU5S_56	0.00	0.00	0.00	0.00	0.00	0.00	0.00	0.00	0.00	0.00
	KU6	Leaf	KU6.4_68	16.03	5.61	0.86	0.71	4.90	2.50	3.18	3.59	0.61	0.89
		Leaf	KU6.5_18	11.37	13.13	1.15	0.56	5.75	0.44	2.60	18.78	0.36	0.46
		Leaf	KU6.6_65	9.52	8.04	0.72	0.32	3.58	0.51	5.29	14.88	0.18	0.35

		Average leaf		12.30 ± 2.74	8.93 ± 3.14	0.91 ± 0.18	0.53 ± 0.16	4.74 ± 0.89	1.15 ± 0.96	3.69 ± 1.16	12.42 ± 6.44	0.39 ± 0.18	0.565 ± 0.23
		Soil	KU6S_12	0.00	0.00	0.00	0.00	0.03	0.00	0.00	0.00	0.00	0.00
MK	MK2	Leaf	MK2.4_03	11.95	10.88	2.65	3.49	1.63	1.22	2.44	3.17	0.29	0.58
		Leaf	MK2.5_34	23.81	20.46	2.70	0.88	2.26	2.14	3.46	4.95	0.06	0.21
		Leaf	MK2.6_08	15.76	14.71	3.00	3.67	0.66	2.36	4.22	2.16	0.26	0.57
		Average leaf		17.17 ± 4.94	15.35 ± 3.94	2.78 ± 0.15	2.68 ± 1.28	1.51 ± 0.66	1.901 ± 0.49	3.37 ± 0.73	3.43 ± 1.15	0.20 ± 0.10	0.45 ± 0.17
		Soil	MK2S_03	0.00	0.01	0.00	0.00	0.19	0.00	0.00	0.00	0.00	0.00
	MK3	Leaf	MK3.4_04	9.60	9.51	2.76	4.92	0.47	1.31	2.49	2.86	0.47	0.85
		Leaf	MK3.5_36	12.54	8.42	2.83	3.35	1.14	1.55	2.08	2.60	2.30	0.71
		Leaf	MK3.6_37	15.25	9.66	1.58	6.84	0.35	2.49	2.33	2.32	0.34	0.62
		Average leaf		12.46 ± 2.31	9.19 ± 0.55	2.39 ± 0.58	5.04 ± 1.43	0.65 ± 0.35	1.78 ± 0.51	2.30 ± 0.17	2.59 ± 0.22	1.04 ± 0.89	0.73 ± 0.09
		Soil	MK3S_34	0.00	0.00	0.00	0.00	0.05	0.00	0.00	0.00	0.00	0.00
	MK4	Leaf	MK4.4_06	14.02	7.52	2.23	2.04	0.62	1.19	1.55	3.04	0.44	1.24
		Leaf	MK4.5_40	10.36	5.89	1.35	2.14	0.97	1.10	1.60	1.40	1.18	1.37
		Leaf	MK4.6_44	10.27	14.46	4.10	1.10	2.32	6.60	4.10	8.04	0.34	0.59
		Average leaf		11.55 ± 1.75	9.29 ± 3.71	2.56 ± 1.15	1.76 ± 0.47	1.30 ± 0.73	2.96 ± 2.57	2.42 ± 1.19	4.16 ± 2.82	0.65 ± 0.37	1.07 ± 0.34
		Soil	MK4S_04	0.00	0.00	0.00	0.00	0.31	0.00	0.00	0.00	0.01	0.02
	MK5	Leaf	MK5.4_07	16.76	23.31	2.79	0.91	0.85	8.36	5.26	3.67	0.53	0.19
		Leaf	MK5.5_41	16.08	21.35	1.38	0.86	1.40	6.49	5.18	4.81	0.07	0.25
		Leaf	MK5.6_45	15.13	14.60	3.87	0.08	2.72	11.85	3.71	6.00	0.27	0.13

		Average leaf		15.99 ± 0.67	19.75 ± 3.73	2.68 ± 1.02	0.62 ± 0.38	1.66 ± 0.79	8.90 ± 2.22	4.72 ± 0.71	4.83 ± 0.96	0.29 ± 0.19	0.19 ± 0.05
		Soil	MK5S_36	0.00	0.00	0.00	0.00	0.09	0.00	0.00	0.00	0.00	0.00
	MK6	Leaf	MK6.4_86	13.97	11.07	2.31	2.76	2.05	0.94	2.94	1.84	0.12	0.77
		Leaf	MK6.5_46	9.75	16.59	6.22	0.02	6.29	3.19	4.43	3.83	0.46	0.48
		Leaf	MK6.6_49	7.43	15.15	5.96	0.63	5.40	5.22	4.73	6.28	0.57	0.37
		Average leaf		10.38 ± 2.71	14.27 ± 2.34	4.83 ± 1.79	1.14 ± 1.17	4.58 ± 1.83	3.12 ± 1.75	4.03 ± 0.78	3.98 ± 1.82	0.38 ± 0.19	0.54 ± 0.17
		Soil	MK6S_37	0.00	0.00	0.00	0.00	0.22	0.00	0.00	0.00	0.00	0.00
	MK9	Leaf	MK9.4_09	12.11	16.23	1.61	1.30	0.50	0.76	4.80	2.91	0.22	0.48
		Leaf	MK9.5_50	18.22	15.07	2.66	0.71	0.87	0.98	4.14	3.86	0.12	0.25
		Leaf	MK9.6_51	20.32	15.65	2.11	1.07	0.32	0.82	4.35	1.57	0.35	0.25
		Average leaf		16.88 ± 3.48	15.65 ± 0.47	2.12 ± 0.43	1.03 ± 0.24	0.56 ± 0.23	0.85 ± 0.09	4.43 ± 0.27	2.78 ± 0.94	0.23 ± 0.10	0.33 ± 0.11
		Soil	MK9S_06	0.00	0.00	0.00	0.00	0.11	0.00	0.00	0.00	0.00	0.00
MV	MV1	Leaf	MV1.4_04	12.96	11.07	0.45	2.15	2.62	3.76	6.43	1.03	1.62	2.53
		Leaf	MV1.6_37	15.44	8.42	0.44	2.86	1.93	4.32	4.27	2.01	1.79	2.31
		Average leaf		14.20 ± 1.24	9.74 ± 1.33	0.45 ± 0.003	2.51 ± 0.36	2.28 ± 0.35	4.04 ± 0.28	5.35 ± 1.08	1.52 ± 0.49	1.71 ± 0.08	2.42 ± 0.11
		Soil	MV1S_46	0.00	0.00	0.00	0.00	0.12	0.00	0.00	0.00	0.00	0.00
	MV2	Leaf	MV2.4_06	19.13	12.38	0.10	1.66	1.60	1.18	4.37	1.54	1.51	2.37
		Leaf	MV2.5_40	21.91	9.09	0.17	1.27	1.69	1.82	4.16	1.49	1.83	2.46
		Leaf	MV2.6_44	20.13	9.16	0.20	1.39	3.07	1.20	4.06	1.31	1.15	2.02
		Average leaf		20.39 ± 1.15	10.21 ± 1.53	0.16 ± 0.04	1.44 ± 0.16	2.12 ± 0.67	1.40 ± 0.30	4.20 ± 0.13	1.45 ± 0.10	1.50 ± 0.28	2.28 ± 0.19

		Soil	MV2S_49	0.00	0.00	0.00	0.00	0.20	0.00	0.00	0.00	0.00	0.00
	MV3	Leaf	MV3.4_07	8.15	15.00	2.01	2.96	2.46	1.34	4.08	4.82	0.19	3.49
		Leaf	MV3.5_41	18.09	14.42	0.58	2.88	1.93	1.24	6.83	1.98	0.43	2.47
		Leaf	MV3.6_45	9.90	15.35	1.18	3.05	2.56	1.81	5.82	0.91	0.73	1.95
		Average leaf		12.05 ± 4.33	14.93 ± 0.39	1.26 ± 0.59	2.97 ± 0.07	2.32 ± 0.28	1.47 ± 0.25	5.58 ± 1.14	2.57 ± 1.65	0.45 ± 0.22	2.64 ± 0.64
		Soil	MV3S_09	0.00	0.01	0.00	0.00	0.14	0.00	0.00	0.00	0.00	0.00
	MV4	Leaf	MV4.4_08	16.68	24.07	0.74	1.95	0.84	3.51	12.69	2.26	0.58	0.95
		Leaf	MV4.5_46	23.82	22.32	0.07	3.00	0.42	2.27	11.17	2.26	0.93	0.83
		Leaf	MV4.6_49	18.84	15.15	0.21	2.55	3.06	2.35	6.93	2.40	0.66	1.38
		Average leaf		19.78 ± 2.99	20.51 ± 3.86	0.34 ± 0.29	2.50 ± 0.43	1.44 ± 1.16	2.71 ± 0.57	10.26 ± 2.44	2.31 ± 0.07	0.72 ± 0.15	1.05 ± 0.23
		Soil	MV4S_50	0.00	0.00	0.00	0.00	0.15	0.00	0.00	0.00	0.00	0.00
	MV5	Leaf	MV5.4_09	14.27	18.26	0.51	2.94	0.82	1.99	8.30	1.48	0.58	1.30
		Leaf	MV5.5_50	17.27	13.89	0.36	4.03	0.90	3.52	6.12	1.39	0.62	0.99
		Leaf	MV5.6_51	15.05	13.00	0.52	2.04	0.98	3.43	5.55	1.24	0.79	1.19
		Average leaf		15.53 ± 1.27	15.05 ± 2.30	0.46 ± 0.07	3.01 ± 0.81	0.90 ± 0.07	2.98 ± 0.70	6.66 ± 1.19	1.37 ± 0.10	0.66 ± 0.09	1.16 ± 0.13
		Soil	MV5S_51	0.00	0.00	0.00	0.00	0.12	0.00	0.00	0.00	0.00	0.00
	MV6	Leaf	MV6.4_10	12.99	13.39	0.14	3.34	1.11	2.12	6.60	0.96	0.38	1.16
		Leaf	MV6.5_55	9.24	20.57	0.14	9.64	1.47	1.68	7.42	1.48	0.50	1.15
		Leaf	MV6.6_53	7.90	11.84	0.07	4.54	1.08	2.67	3.01	0.54	0.31	1.91
		Average leaf		10.04 ± 2.15	15.26 ± 3.80	0.12 ± 0.03	5.84 ± 2.73	1.22 ± 0.18	2.15 ± 0.41	5.68 ± 1.92	0.99 ± 0.39	0.39 ± 0.08	1.41 ± 0.36
		Soil	MV6S_10	0.00	0.00	0.00	0.00	0.19	0.00	0.00	0.00	0.00	0.00

SL	SL1	Leaf	SL1.4_10	21.35	3.27	0.85	0.51	4.16	0.27	0.57	2.30	3.86	1.27
		Leaf	SL1.5_55	2.31	8.08	0.66	0.04	13.56	0.03	1.83	5.54	7.39	1.47
		Leaf	SL1.6_53	5.20	5.25	0.83	0.27	5.65	0.19	0.74	1.30	3.76	1.29
		Average leaf		9.62 ± 8.38	5.53 ± 1.97	0.78 ± 0.09	0.27 ± 0.19	7.79 ± 4.13	0.16 ± 0.10	1.05 ± 0.56	3.04 ± 1.81	5.00 ± 1.69	1.34 ± 0.09
		Soil	SL1S_40	0.00	0.00	0.00	0.00	0.11	0.00	0.00	0.00	0.00	0.00
	SL2	Leaf	SL2.4_11	1.56	4.82	0.12	0.05	22.40	0.09	0.67	2.27	2.94	0.15
		Leaf	SL2.5_58	0.48	3.14	0.20	0.05	22.55	0.03	0.52	5.83	3.43	0.05
		Leaf	SL2.6_56	0.96	3.34	0.36	0.10	17.61	0.09	0.36	14.98	3.77	0.17
		Average leaf		1.00 ± 0.44	3.77 ± 0.75	0.23 ± 0.10	0.07 ± 0.03	20.86 ± 2.30	0.07 ± 0.03	0.52 ± 0.13	7.69 ± 5.36	3.38 ± 0.34	0.12 ± 0.06
		Soil	SL2S_44	0.00	0.00	0.00	0.00	0.06	0.00	0.00	0.00	0.00	0.00
	SL3	Leaf	SL3.4_12	10.55	6.44	1.87	0.18	12.15	0.33	0.52	7.46	5.06	0.88
		Leaf	SL3.5_60	3.62	10.67	1.00	0.16	22.03	0.13	1.04	3.75	4.66	0.87
		Leaf	SL3.6_57	37.17	2.16	1.82	0.16	3.51	0.06	0.21	2.47	4.96	1.17
		Average leaf		17.11 ± 14.46	6.42 ± 3.47	1.56 ± 0.40	0.17 ± 0.01	12.56 ± 7.57	0.17 ± 0.12	0.59 ± 0.34	4.56 ± 2.12	4.89 ± 0.17	0.97 ± 0.14
	SL4	Leaf	SL4.4_14	10.84	11.94	0.66	0.15	13.33	0.25	1.26	4.57	3.39	0.72
		Leaf	SL4.5_61	1.10	6.62	0.61	0.07	16.19	0.15	0.44	4.04	4.85	0.78
		Leaf	SL4.6_05	0.85	3.87	1.05	0.13	15.13	0.41	0.55	2.05	4.24	0.63
		Average leaf		4.26 ± 4.65	7.48 ± 3.35	0.77 ± 0.20	0.12 ± 0.03	14.89 ± 1.18	0.27 ± 0.11	0.75 ± 0.37	3.55 ± 1.08	4.16 ± 0.60	0.71 ± 0.06
		Soil	SL4S_41	0.00	0.00	0.00	0.00	0.02	0.00	0.00	0.00	0.00	0.00
	SL7	Leaf	SL7.4_13	22.67	6.31	1.24	0.42	4.94	0.04	0.95	9.77	4.90	0.69

		Leaf	SL7.5_66	19.71	4.98	1.23	0.42	6.13	0.06	0.77	3.50	4.15	1.34
		Leaf	SL7.6_81	1.74	4.64	0.48	0.16	13.31	0.14	0.49	4.43	5.38	0.59
		Average leaf		14.71 ± 9.25	5.31 ± 0.72	0.98 ± 0.36	0.34 ± 0.13	8.13 ± 3.70	0.08 ± 0.04	0.74 ± 0.19	5.90 ± 2.76	4.81 ± 0.51	0.87 ± 0.33
		Soil	SL7S_45	0.00	0.00	0.00	0.00	0.00	0.00	0.00	0.00	0.00	0.00
	SL8	Leaf	SL8.4_85	1.73	1.85	0.32	0.15	20.53	0.02	0.34	19.34	1.35	0.16
		Leaf	SL8.5_03	12.75	4.41	1.63	0.91	8.51	0.56	1.15	4.84	3.93	1.15
		Leaf	SL8.6_34	5.36	4.51	0.42	0.20	14.36	0.05	0.28	5.85	3.06	0.95
		Average leaf		6.62 ± 4.59	3.59 ± 1.23	0.79 ± 0.60	0.42 ± 0.35	14.47 ± 4.91	0.21 ± 0.25	0.59 ± 0.40	10.01 ± 6.61	2.78 ± 1.07	0.75 ± 0.43
		Soil	SL8S_08	0.00	0.00	0.00	0.00	0.00	0.00	0.00	0.00	0.00	0.00
HT site average (leaf)				8.40 ± 4.63	9.41 ± 4.67	0.64 ± 0.92	4.70 ± 3.80	1.35 ± 1.55	0.54 ± 0.32	3.27 ± 1.86	2.08 ± 1.53	0.16 ± 0.19	0.61 ± 0.48
KU site average (leaf)				11.45 ± 5.57	8.60 ± 3.32	0.66± 0.34	1.13 ± 0.66	5.10 ± 3.49	1.10 ± 0.75	2.69 ± 1.41	6.10 ± 5.09	0.59 ± 0.33	0.79 ± 0.46
MK site average (leaf)				14.07 ± 4.01	13.92 ± 4.69	2.89 ± 1.35	2.04 ± 1.77	1.71 ± 1.63	3.25 ± 3.08	3.55 ± 1.18	3.63 ± 1.74	0.47 ± 0.51	0.55 ± 0.34
MV site average (leaf)				15.40 ± 4.63	14.55 ± 4.39	0.46 ± 0.48	3.08 ± 1.85	1.68 ± 0.81	2.37 ± 0.97	6.34 ± 2.48	1.71 ± 0.93	0.86 ± 0.51	1.79 ± 0.72
SL site average (leaf)				8.89 ± 9.99	5.35 ± 2.62	0.85 ± 0.52	0.23 ± 0.21	13.12 ± 6.29	0.16 ± 0.15	0.71 ± 0.40	5.79 ± 4.55	4.17 ± 1.22	0.80 ± 0.43

Relative abundance of core taxa in phyllosphere samples is averaged per tree and site (mean ± SD).
