# Supplementary material for: A core phyllosphere microbiome exists across distant populations of a tree species indigenous to New Zealand
Source: PLoS One. 2020 Aug 13;15(8):e0237079. doi: 10.1371/journal.pone.0237079 (PMC7425925; doi:10.1371/journal.pone.0237079)
Supplement: S9 Table — Correlations are performed on Bray Curtis dissimilarity of total, non-core, and core taxa and Euclidean distances of spatial parameters using Pearson’s product-moment correlation coefficient test (Corr). Positive correlations greater than 0.5 are underlined. (PDF) [file pone.0237079.s020.pdf]

S9 Table: Mantel and Partial Mantel test correlations between mānuka phyllosphere community dissimilarity and spatial variables.

	Total		Non-core		Core	
	Corr	*P test*	*Corr*	*P test*	*Corr*	*P test*
Distance	0.15	0.01	0.27	0.002	-0.05	0.69
Latitude	0.24	0.01	0.24	0.007	0.26	0.02
Longitude	0.33	0.001	0.36	0.001	0.17	0.02
Elevation	<u>0.58</u>	0.001	<u>0.60</u>	0.001	0.43	0.001

Correlations are performed on Bray Curtis dissimilarity of total, non-core, and core taxa and Euclidean distances of spatial parameters using Pearson's product-moment correlation coefficient test (Corr). Positive correlations greater than 0.5 are underlined.
