# Supplementary material for: A core phyllosphere microbiome exists across distant populations of a tree species indigenous to New Zealand
Source: PLoS One. 2020 Aug 13;15(8):e0237079. doi: 10.1371/journal.pone.0237079 (PMC7425925; doi:10.1371/journal.pone.0237079)
Supplement: S10 Table — Correlations are performed on Bray Curtis dissimilarity of total, non-core, and core taxa and Euclidean distances of environmental parameters using Pearson’s product-moment correlation coefficient test (Corr). Positive correlations greater than 0.5 are underlined. The symbol (m) represents monthly averages obtained from the NIWA database. Other environmental conditions were measured with a datalogger at each site every 15-minute intervals across the 24 h period prior to sampling. (PDF) [file pone.0237079.s021.pdf]

S10 Table: Partial Mantel test correlations between mānuka phyllosphere community dissimilarity and Euclidean distances of environmental variables.

	Total		No core		Core	
	Corr	*P test*	*Corr*	*P test*	*Corr*	*P test*
Day temperature	0.23	0.003	0.21	0.003	0.11	0.04
Night Temperature	<u>0.78</u>	0.001	<u>0.80</u>	0.001	<u>0.63</u>	0.001
Day/night temperature differential	<u>0.65</u>	0.001	<u>0.62</u>	0.001	0.49	0.001
Relative humidity	0.17	0.001	0.11	0.03	0.11	0.05
Photosynthetically active radiation	-0.01	0.56	-0.08	0.87	-0.02	0.60
Temperature (m)	-0.01	0.42	0.02	0.35	0.06	0.23
Precipitation (m)	<u>0.58</u>	0.001	<u>0.62</u>	0.001	0.44	0.001
Wind (m)	-0.07	0.75	-0.06	0.77	0.01	0.39
Pressure (m)	-0.16	0.011	0.14	0.02	0.08	0.06
Cloud (m)	<u>0.60</u>	0.001	<u>0.60</u>	0.001	0.41	0.001
Humidity (m)	-0.39	0.001	0.38	0.001	0.22	0.02
Sun (m)	<u>0.61</u>	0.001	<u>0.61</u>	0.001	0.37	0.001

Correlations are performed on Bray Curtis dissimilarity of total, non-core, and core taxa and Euclidean distances of environmental parameters using Pearson's product-moment correlation coefficient test (Corr). Positive correlations greater than 0.5 are underlined. The symbol (m) represents monthly averages obtained from the NIWA database. Other environmental conditions were measured with a datalogger at each site every 15-minute intervals across the 24 h period prior to sampling.
