# Supplementary material for: A core phyllosphere microbiome exists across distant populations of a tree species indigenous to New Zealand
Source: PLoS One. 2020 Aug 13;15(8):e0237079. doi: 10.1371/journal.pone.0237079 (PMC7425925; doi:10.1371/journal.pone.0237079)
Supplement: S11 Table — Correlations are performed on Bray Curtis dissimilarity of total, non-core, and core taxa and Euclidean distances of host tree parameters using Pearson’s product-moment correlation coefficient test (Corr). (PDF) [file pone.0237079.s022.pdf]

S11 Table: Partial Mantel test correlations between mānuka phyllosphere community dissimilarity and Euclidean distances of host variables.

	Total		No core		Core	
	Corr	*P test*	*Corr*	*P test*	*Corr*	*P test*
Branch height	-0.06	0.90	-0.08	0.97	-0.06	0.88
Branch aspect	-0.01	0.56	-0.01	0.56	-0.003	0.51
Tree height	0.19	0.02	0.23	0.004	0.12	0.10
Tree diameter	-0.04	0.59	-0.06	0.73	-0.02	0.53

Correlations are performed on Bray Curtis dissimilarity of total, non-core, and core taxa and Euclidean distances of host tree parameters using Pearson's product-moment correlation coefficient test (Corr).
