# Supplementary material for: A core phyllosphere microbiome exists across distant populations of a tree species indigenous to New Zealand
Source: PLoS One. 2020 Aug 13;15(8):e0237079. doi: 10.1371/journal.pone.0237079 (PMC7425925; doi:10.1371/journal.pone.0237079)
Supplement: Attachment — Submitted filename: Response to Reviewers.docx [file pone.0237079.s023.docx]

**Comments from the Editor**

1. **Comment:** Please ensure that your manuscript meets PLOS ONE's style requirements, including those for file naming. The PLOS ONE style templates can be found at http://www.plosone.org/attachments/PLOSOne_formatting_sample_main_body.pdf and http://www.plosone.org/attachments/PLOSOne_formatting_sample_title_authors_affiliaaffil.pdf

**Author response:** We have revised the manuscript and associated files to meet the PLOS ONE style requirements.

**2. Comment:** In your Methods section, please provide additional information regarding the permits you obtained for the work. Please ensure you have included the full name of the authority that approved the collections sites access and, if no permits were required, a brief statement explaining why.

**Author response:** We now include the full names of the authorities that approved collection site access. The revised text reads as follows:

Lines 158-161: *“Steens Honey provided access to the sites in Mohaka and Mangatarere Valley. The East Taupō Lands Trust provided access to the north-eastern Kaimanawa site. Timberlands Ltd provided access to the Mamaku site. The Waipa District Council provided access to the site at Serpentine Lake.”*

**3. Comment:** We note that you are reporting an analysis of a microarray, next-generation sequencing, or deep sequencing data set. PLOS requires that authors comply with field-specific standards for preparation, recording, and deposition of data in repositories appropriate to their field. Please upload these data to a stable, public repository (such as ArrayExpress, Gene Expression Omnibus (GEO), DNA Data Bank of Japan (DDBJ), NCBI GenBank, NCBI Sequence Read Archive, or EMBL Nucleotide Sequence Database (ENA)). In your revised cover letter, please provide the relevant accession numbers that may be used to access these data. For a full list of recommended repositories, see http://journals.plos.org/plosone/s/data-availability#loc-omics or http://journals.plos.org/plosone/s/data-availability#loc-sequencing.

**Author response:** The sequence reads are available in the NCBI Sequence Read Archive (SRA) under the accession number PRJNA556542 as indicated in the “Data availability” section in the manuscript.

4. **Comment:** We note that Figure S1 in your submission contain map images which may be copyrighted. All PLOS content is published under the Creative Commons Attribution License (CC BY 4.0), which means that the manuscript, images, and Supporting Information files will be freely available online, and any third party is permitted to access, download, copy, distribute, and use these materials in any way, even commercially, with proper attribution. For these reasons, we cannot publish previously copyrighted maps or satellite images created using proprietary data, such as Google software (Google Maps,

Street View, and Earth). For more information, see our copyright guidelines: http://journals.plos.org/plosone/s/licenses-and-copyright. We require you to either (1) present written permission from the copyright holder to publish these figures specifically under the CC BY 4.0 license, or (2) remove the figures from your submission:

**Author response:** We have provided a replacement Figure S1 that uses data sourced from the Natural Earth public domain and complies with the CC BY 4.0 license. The new figure caption reads as follows:

***“S1 Fig: Location of Leptospermum scoparium (mānuka) populations in the North Island of New Zealand.*** *Borders represent regional boundaries. Total rain (mm) and total sun (mm) data retrieved from the National Climate Database (NIWA) for the month prior to sampling. Map made with Natural Earth.”*

**Comments from Reviewer 1**

The authors investigated microbial communities on five Mānuka populations in New Zealand using 16S amplicon sequencing. They contrasted the phyllosphere community composition from the composition of surface soil underneath the trees.

The english used in the publication is good and the sequencing and formal analysis is of high standard.

1. The claimed hypothesis that antimicrobial activity of honey is related to the phyllosphere microbiome cannot be answered by the analysis and experiments performed. If the authors were to address this question, they would have to measure the activity of honey associated with individual plants and determine the phyllosphere microbiome of those plants too.

**Author response:** We thank the reviewer for raising this point and apologise for not highlighting our testable hypothesis more explicitly in the original manuscript. Our broader research is underpinned by a two-part hypothesis: 1) the mānuka phyllosphere microbiome plays a role in mediating host physiology, and therefore 2) the mānuka phyllosphere microbiome exhibits specific host association. We acknowledge that only the latter was testable within the context of the analyses included in our manuscript. We have clarified our testable hypothesis and its corresponding null hypothesis in the revised manuscript. These changes can be found in the abstract and the introduction and read as follows:

Lines 20-26: *“Notably, the mānuka phyllosphere microbiome remains uncharacterised, and its potential role in mediating host physiology has not been considered. Working within the prevailing core microbiome conceptual framework, we hypothesise that the phyllosphere microbiome of mānuka has a role in mediating host physiology and thus exhibits specific host association patterns congruent with those of a phyllosphere*

*microbiome that mediates host physiology (null hypothesis: the mānuka phyllosphere microbiome is recruited stochastically from the surrounding environment).”*

Lines 131-150: “*Inspired by the unexplained heterogeneities in mānuka DHA production and the emerging role of the phyllosphere microbiome in plant physiology, we intuit that the phyllosphere microbiome may have a significant yet unevaluated role in mediating mānuka physiology. Working within the prevailing core microbiome conceptual framework, we hypothesise that phyllosphere microorganisms of mānuka exhibit specific host association patterns congruent with those of a phyllosphere microbiome that mediates host physiology. Correspondingly, our null hypothesis is that the mānuka phyllosphere microbiome is recruited stochastically from the surrounding environment. To test this hypothesis, we characterised the phyllosphere and associated soil bacterial communities of five distinct and geographically distant mānuka populations across the North Island of New Zealand using 16S rRNA gene PCR amplicon sequencing. Our findings revealed a dominant and ubiquitous core microbiome, providing strong evidence for a specific host association and thus potentiating the role of the phyllosphere microbiome in influencing mānuka physiology. A better understanding of the mānuka phyllosphere microbiome will enhance knowledge regarding plant-microbe associations in the phyllosphere and address significant and longstanding questions such as the factors driving spatiotemporal variability in mānuka DHA production.”*

2. For arguments sake, why would antimicrobial activity be related to phyllosphere microbes compared to endophytes or the rhizosphere microbiome? This is not addressed at all.

**Author response:** Our hypotheses (see our response to comment #1 where we clarify our testable and null hypotheses) are largely driven by recent evidence that suggests the phyllosphere microbiome has an underappreciated role in the functioning of their host plants. Such evidence has shown that the phyllosphere microbiome plays a key role in (1) plant adaptation to abiotic stressors such as frost (Cid et al., 2018 [5]) and drought (Kumaravel et al., 2018 [6]), (2) plant defence against pathogens (Innerebner et al., 2011 [69]), (3) plant productivity through the synthesis of phytohormones (Glickmann et al., 1998) and cytokinins (Brandl and Lindow, 1998), (4) terrestrial ecosystem productivity (Laforest-Lapointe et al., 2017) and nutrient cycling (Furnkranz et al., 2008 [70]). Given the phyllosphere microbiome has the potential to influence various aspects of host plant physiology but not been previously studied in mānuka (the rhizosphere and endosphere have been investigated), we postulated that investigating the mānuka phyllosphere microbiome could be of crucial importance. We have amended the text within the introduction to better outline our rationale:

Lines 41-51: *“Plants harbour distinct and dynamic environments colonised by complex microbial communities known as host microbiomes [1]. Evidence has shown that interactions between plants and their associated microorganisms in many of these microbiomes play a pivotal role in host plant health, function, and evolution [2]. The leaf surface, or phyllosphere, harbours a microbiome comprising diverse communities of bacteria, fungi, algae, archaea, and viruses [3]. Interactions between the host plant*

*and phyllosphere bacteria have the potential to drive various aspects of host plant physiology [4-6]. However, our knowledge of these bacterial associations in the phyllosphere remains relatively modest, and there is a need to advance fundamental knowledge of phyllosphere microbiome dynamics [7].”*

Lines 111-130: “*Leptospermum scoparium var. scoparium (Myrtaceae), commonly known as “mānuka”, is a flowering tea tree indigenous to New Zealand [34]. Mānuka honey, produced from the nectar of mānuka flowers, is globally renowned for its unique non-peroxide antibacterial properties [35, 36]. These non-peroxide antibacterial properties have been principally linked to the accumulation of the three-carbon sugar dihydroxyacetone (DHA) in the nectar of the mānuka flower, which undergoes a chemical conversion to methylglyoxal (MGO) in mature honey [37-39]. However, the concentration of DHA in the nectar of mānuka flowers is notoriously variable, and the antimicrobial efficacy of mānuka honey consequently varies from region to region and from year to year [40-42]. Despite extensive research efforts, no reliable correlation has been identified between DHA production and climatic [43], edaphic [44], or host genetic factors [45]. Microorganisms have been studied in the mānuka rhizosphere and endosphere [46-48]. Although existing studies have primarily focussed on fungi, a recent study provided the first investigation of endophytic bacterial communities from three geographically and environmentally distinct mānuka populations using fingerprinting techniques and revealed tissue-specific core endomicrobiomes [49]. However, the role of microorganisms in driving DHA variation remain unknown, and a characterisation of the mānuka phyllosphere microbiome has not been conducted.”*

Lines 131-138: *“Inspired by the unexplained heterogeneities in mānuka DHA production and the emerging role of the phyllosphere microbiome in plant physiology, we intuit that the phyllosphere microbiome may have a significant yet unevaluated role in mediating mānuka physiology. Working within the prevailing core microbiome conceptual framework, we hypothesise that phyllosphere microorganisms of mānuka exhibit specific host association patterns congruent with those of a phyllosphere microbiome that mediates host physiology.”*

3. In a nutshell, I believe that the authors should reformulate their hypothesis and question so that they can actually test them with their dataset. I suggest to focus on geographical location and latitude instead.

**Author response:** We thank the reviewer for the suggestion and acknowledge that the way our testable hypothesis was presented and justified in the original manuscript required further clarification. We hope that the revisions made in response to comment #1 have improved the clarity of our study’s testable hypothesis.

4. I find that the argument that a core microbiome would be responsible for the medicinal/ antimicrobial activity flawed. Wouldn’t fluctuations of this activity better be explained by the changes in accessory microbiota?

**Author response:** We thank the reviewer for bringing up this question. We would like to reassure the reviewer that it was not our intention to imply that the core microbiome (if

present) is responsible for the medicinal/antimicrobial properties of the host plant. Rather, we argue that the presence of a core microbiome is indicative of specific host association patterns (please refer to our response to comment #1 where we have clarified our testable and null hypotheses). We apologise for giving the reviewer this incorrect impression.

The search for a core microbiome in host-associated habitats is regarded by many as a useful first step in trying to understand the dynamics of the interactions that may be occurring between a host and its microbiome (NB: please also see the response to comment #5 where we highlight the inconsistency in how “core microbiome” has been defined across scientific literature). Given the interesting yet unexplained physiological characteristics of mānuka, we respectfully maintain that searching for signs of strong and specific host association is a useful top-down approach to identify whether its phyllosphere microbiome might interact with the host. We identified a core microbiome at the OTU level, which validates our testable hypothesis and serves as a robust starting point to begin testing the other part of our two-part hypothesis (please refer to the response to comment #1).

We also argue that members of a prominent core microbiome can plausibly be responsible for heterogeneities in host physiology since species/strains within individual OTUs (defined at 97% similarity in partial rRNA gene sequence for our study) are not necessarily functionally homogeneous (as reviewed in VanInsberghe et al., 2020 [71], doi:10.1098/rstb.2019.0253). Recent studies also show host-microbe functional outcomes occur at the level of intraspecific variants (For example, Costea et al., 2017, doi: 10.15252/msb.20177589). In other words, a core microbiome at the OTU level may exhibit large functional diversity across hosts and thus effect divergent host physiological responses. We have elaborated on the functional potential of the core microbiome in the discussion to further clarify these ideas:

Lines 518-535: *“In accordance with the prevailing core microbiome concept, a stable association between the mānuka host and a habitat-specific group of microorganisms suggests these taxa are functionally important for the integrity of the phyllosphere microbiome and host plant [29, 30]. Consistent with this notion, multiple members affiliated with the core bacterial genera identified here are known to associate closely with plants. For example, Sphingomonas has been shown to promote host plant health by reducing the growth of pathogenic Pseudomonas strains [69]. Rhizobiales also contains many genera of diazotrophic and methylotrophic bacteria that drive important processes in the phyllosphere, and provide various nutrients, phytohormones, and precursors for essential plant metabolites [51, 70]. However, given the large strain-level functional diversity of microorganisms and that many members of the core mānuka phyllosphere microbiome are distantly related to their closest characterised matches, the functional roles of these core taxa remain an open question [71].”*

5. As a general note, the use of references is excessive - not every fact needs three references in the introduction. Also in the introduction, the authors go back and forth between mammalian and plant references a lot - since this is a study relating to plants and all the concepts they are referring too are well established, they should focus on those rather than mammalian studies. E.g. the concept of core microbiome is well

studied in the phyllosphere. This will streamline and focus the study while avoiding overstatements.

**Author response:** We thank the reviewer for pointing this out. We have carefully considered all the citations and removed a number of unnecessary ones that referred to the identification of mammalian core microbiomes. Contrary to the reviewer’s comment, however, our review of current literature indicated that the core microbiome concept is relatively poorly established for the phyllosphere and requires careful review and clarification. In part, this has resulted from inconsistencies in how the term “phyllosphere” is defined across literature (see our response to comment #10 where we justify our definition of phyllosphere). This has also largely resulted from inconsistencies in how the term “core microbiome” is defined across scientific literature, and currently no consensus has been reached in any of the organisms to which the concept has been applied (the implications of this with respect to the coral core microbiome has been reviewed by Hernandez-Agreda et al. 2017 [33], doi: 10.1016/j.tim.2016.11.003). For example, researchers have variably identified “core taxa” as those persistent across distinct host microhabitats and even different species. However, microorganisms are functionally divergent across both different host species and microhabitats, thus ubiquitous microorganisms identified in this approach are unlikely to represent specific host associations. Furthermore, spatial scale must be carefully considered in core microbiome analyses. For example, identifying taxa persistent within a single host population may reflect positive selection by the host or transient microorganisms dispersed from the surrounding environment. Identifying taxa persistent across multiple discrete host populations are more likely to reflect positive selection by the host (i.e., specific host association) since each host population is unlikely to be exposed to the same transient microorganisms via passive dispersal. Thus, core analyses across small spatial scales provide a different, and arguably less ecologically relevant, core than core analyses across large geographic distances. Within this context, we therefore assert that defining core taxa *sensu stricto* as those persistent across broad geographical distances within tissue- and species-specific host microbiomes represents the most biologically and ecologically relevant application of this framework. To our knowledge, tissue- and species-specific core microbiomes across host populations separated by broad geographical distances have not been widely reported for the phyllosphere and warrant a systematic investigation like ours. We apologise for not making this clearer with the original manuscript and have made changes to streamline and clarify our argument.

Lines 78-99: *“The search for a core microbiome in host-associated microbial communities is a useful first step in trying to understand the interactions that may be occurring between a host and its microbiome [29]. The prevalent core microbiome concept is built on the notion that the persistence of a taxon across the spatiotemporal boundaries of an ecological niche is directly reflective of its functional importance within the niche it occupies and therefore provides a framework for identifying functionally critical microorganisms that consistently associate with a host species [29-31]. However, divergent definitions of “core microbiome” have arisen across scientific literature with researchers variably identifying “core taxa” as those persistent across distinct host microhabitats [19, 32] and even different species [24, 28]. Here we assert*

*that, given the functional divergence of microorganisms across different host species [24] and microhabitats [12], defining core taxa sensu stricto as those persistent across broad geographic distances within tissue- and species-specific host microbiomes, represents the most biologically and ecologically appropriate application of this conceptual framework [33]. To our knowledge, tissue- and species-specific core microbiomes across host populations separated by broad geographical distances have not been widely reported for the phyllosphere.”*

6. As another general note - many references are not appropriately chosen and should be double checked - by just looking into a few I found many that were not cited in a correct context. Going through all is too big of a job for this already extensive review.

**Author response:** We apologise for errors in our referencing. We have made changes to the references specifically mentioned in comments #19, #21, and #22. We have also carefully considered all other citations and removed a number of unnecessary ones that referred to core microbiomes in mammals (as mentioned in the response to comment #5).

7. The introduction could be streamlined by reorganising the lines of thoughts. Currently, it goes back and forth between different lines of thoughts. The authors will have to do their due diligence and carefully check everything themselves.

**Author response:** We have revised the manuscript with this critique in mind, and we hope that our revisions made in response to comments #1, #2, #5, and #10 have streamlined the focus of the introduction.

8. Lines 51-55 I think these are big words - they need more explanation and references. “Must” is imperative and it is unclear to me what the authors are referring too when they assert that this “must” be done. Plant leaves and other plant organs have previously been shown to exude nutrients. Plant leaves have species specific physicochemistries and morphologies etc. The existents of a core microbiome does not necessarily relate to host-microbe interactions, even though it is tempting to speculate the latter.

**Author response:** We hope the revisions made in response to comment #5 (which include the original lines 51-55) have clarified the usefulness of the core microbiome concept as a *framework* with which top-down cultivation-independent studies can identify specific host association.

9. Drivers of plant microbiotas have been reviewed elsewhere - I suggest the authors use these reviews to support their claims.

**Author response:** We thank the reviewer for the suggestion and have included a number of relevant reviews within the manuscript. The reviews we included are:

3. Leveau JHJ. A brief from the leaf: latest research to inform our understanding of the phyllosphere microbiome. Current Opinion in Microbiology. 2019;49:41-9. doi: https://doi.org/10.1016/j.mib.2019.10.002.

7. Laforest-Lapointe I, Whitaker BK. Decrypting the phyllosphere microbiota: progress and challenges. American Journal of Botany. 2019;106(2):171-3. doi: 10.1002/ajb2.1229.

10. Line 56 The phyllosphere is often troublesome to define and different definitions can be found - refer to the 2019 review of Johan Leveau. Most often it has been defined as the microbial habitat that encompasses the above-ground surface of plants and is contrasted by the rhizosphere.

**Author response:** We absolutely agree with the reviewer that the term ‘phyllosphere’ has been used inconsistently across literature, including a broad inclusion of all microbial communities colonising both the inside and surface of all aerial plant structures. This broad definition (and its subsets) encompasses numerous distinct microbial habitats that harbour specialised and functionally diverse microbial communities, making it deeply problematic for microbial ecology research where habitats should be defined and described at scales relevant to microorganisms. Therefore, we used the original definition of ‘phyllosphere’ (Ruinen 1956 [20]) to refer strictly to the leaf surface and all the microorganisms that reside on it. We agree that this is important to clarify in our manuscript and have revised the text to emphasise our focus on leaf epiphytes and how we choose to define the term “phyllosphere”. The revised text reads as follows:

Lines 52-64: *“One of the major factors limiting our understanding of these bacterial associations is the inconsistency in how the phyllosphere is defined in the scientific literature [8, 9]. In the broadest sense, the term phyllosphere has been used to refer to the epiphytic and endophytic microbial communities inhabiting all aerial plant structures [10, 11]. This broad definition encompasses numerous distinct plant microhabitats that harbour specialised and functionally diverse microbial communities [12], making it deeply problematic for microbial ecology research where habitats must be defined and described at microbiologically relevant scales [13, 14]. The term phyllosphere has also been used to describe epiphytic microorganisms on aerial plant structures [15, 16], as well as the epiphytic and endophytic communities of leaves [17-19]. Here, we adopt the original and stringent definition of the term ‘phyllosphere’ to refer exclusively to epiphytic communities on the leaf surface, which is generally considered a discrete microbial habitat [9, 20, 21].”*

11. The authors spend many words on core-microbiome while also stating that conflicting information exists for some plant hosts. Doesn’t that already suggest that the concept as the authors want to use it does not hold? This seems like a circular argument. It has been claimed before that the phyllosphere microbiome of plants is compositionally consistent at a low phylogenetic level - i.e. different species of the same genus can be found in different environments and in different years. Will this change you assessment in lines 61-66?

**Author response:** We agree that the phyllosphere microbiome for some plants appears compositionally consistent. For example, Redford et al. 2010 [9] identified fundamentally similar phyllosphere communities in pine trees located in different continents. However, not all phyllosphere microbiomes are compositionally consistent, and some appear shaped by neutral processes of community assembly. For example, Finkel et al., 2012 [26] showed that dispersal limitation plays a role in structuring the phyllosphere microbiome of the salt secreting *Tamarix* tree. Furthermore, the grape vine exhibits significant regional variation in phyllosphere community composition, suggested to be driven by the soil microbiome (Zarraonaindia et al., 2015 [25]). Because of this conflicting evidence, the processes that drive phyllosphere community assembly are not well understood, and we cannot assume the phyllosphere of any given plant species will be compositionally consistent at the OTU level. We have clarified this within the introduction:

Lines 65-77: *“The assembly of the phyllosphere microbiome can be shaped by the microbial communities present in the surrounding environment (i.e., stochastic colonisation) and the host plant (i.e., biotic selection) [3, 22]. However, there is no consensus on the dominant driver of community assembly across phyllosphere microbiomes. For example, host-specific bacterial communities have been reported in the phyllosphere of co-occurring plant species, suggesting a dominant role of host selection [9, 23, 24]. Conversely, microbiomes of the surrounding environment have also been reported to be the primary determinant of phyllosphere community composition [21, 25-27]. As a result, the processes that drive phyllosphere community assembly are not well understood but unlikely to be universal across plant species. However, the existing evidence does indicate that phyllosphere microbiomes exhibiting host-specific associations are more likely to interact with the host than those primarily recruited from the surrounding environment [9, 24, 28].”*

Given the inconsistency in how both terms “core microbiome” and “phyllosphere” are defined across scientific literature (see response to comment #5 where we explain how these inconsistencies affect the ecological relevance of defined core microbiota), as well as the variability evident in the phyllosphere microbiome, it is unclear whether plants harbour core microbiomes as per our definition. This topic therefore warrants further systematic investigation like our study.

12. The authors missed to cite the study of Wicaksono et al. (10.1371/journal.pone.0163717) who were in my knowledge the first who investigate the Mānuka endosphere microbiome. The here presented study should be compared and contrasted with this previous study.

**Author response:** We thank the reviewer for pointing this out and apologise for our failure to cite this important study. We have now cited and described the Wicaksono et al. study [49] in the introduction. This reads as follows:

Lines 124-130: “*Microorganisms have been studied in the mānuka rhizosphere and endosphere [46-48]. Although existing studies have primarily focussed on fungi, a recent study provided the first investigation of endophytic bacterial communities from three geographically and environmentally distinct mānuka populations using fingerprinting techniques and revealed tissue-specific core endomicrobiomes [49].*

*However, the role of microorganisms in driving DHA variation remain unknown, and a characterisation of the mānuka phyllosphere microbiome has not been conducted.”*

13. Next to the study above - it is not discussed how unique the mānuka microbiota is - please compare and contrast from other phyllosphere studies.

**Author response:** We thank the reviewer for pointing this out. We have made significant changes within our discussion that now compares and contrasts the mānuka phyllosphere microbiome with other phyllosphere studies.

Lines 489-517: *“The core phyllosphere microbiome (10 OTUs) represented a large proportion of the total phyllosphere community yet was either rare or entirely absent in surrounding surface soil, suggesting specialisation of these microbial taxa to the habitat or strong selection by the host plant. At the phylum level, the core microbiome comprised major lineages of bacteria also identified in the phyllosphere of other plant species [9, 24, 27]. Four of the most abundant core OTUs were affiliated with the Rhizobiales order (Alphaproteobacteria). OTUs related to order Rhizobiales have frequently been identified in the phyllosphere of temperate forest tree species [65, 66]. Other core taxa were assigned to genera not commonly reported in the phyllosphere, such as Terriglobus, and many core OTUs represented previously undescribed microorganisms, indicating a diversity within these phyllosphere bacterial lineages that may be unique to mānuka. The apparent host-specific association in the mānuka phyllosphere microbiome supports previous studies that identified species-specific communities in the phyllosphere of co-occurring plant species [24]. Genotype-dependent characteristics of leaf morphology and chemistry have been suggested to drive such host-specific patterns [28]. Notably, medicinal plants have previously been reported to harbour highly specialised microbial communities due to their unique and structurally divergent secondary metabolites [67]. The unique components of mānuka’s antimicrobial essential oils may therefore play a role in the selection and maintenance of specific bacterial lineages in its phyllosphere across large geographical ranges and variable environments [68].”*

Lines 563*-579: “Significant differences in community composition were observed between the host microbiome in the phyllosphere and free-living communities in the surrounding soil. As expected, soil bacterial communities were more diverse than those in the phyllosphere. Given the environmental heterogeneity of the surrounding environments in which we sampled, the collection of neutral and deterministic forces shaping the soil communities were likely very diverse. Notably, taxa that were abundant in the phyllosphere, including the core microbiome, were essentially absent in surrounding soil. This finding contrasts with previous studies that report a large proportion of abundant phyllosphere taxa are also present in soil [25, 79]. Although the major reservoir of the mānuka phyllosphere microbiome cannot be directly inferred from our analysis, if phyllosphere bacterial communities were assembled stochastically from the surrounding environment, the diversity of the surrounding soils would have been expected to drive large regional differences as observed in other plant species, such as the grape vine [25]. Instead, the convergence consistently observed in the phyllosphere microbiome independent of the surrounding environment suggests that*

*soil has limited influence on the community structure of the mānuka phyllosphere microbiome.”*

14. I am surprised that top soil microbiome was used to contrast the phyllosphere samples - this is well known to be distinct from leaf microbiomes and rhizosphere microbiomes. This does not seem a suitable comparison. Instead, other plants in the surrounding would have been useful.

**Author response:** Previous studies have suggested the phyllosphere microbiomes of certain plants (e.g., grape vine in Zarraonaindia et al., 2015 [25]) are recruited from free-living microbial communities in surrounding soil. To test our null hypothesis that the mānuka phyllosphere microbiome is recruited stochastically from the surrounding environment (please refer to our revised hypotheses in the response to comment #1), we therefore decided to compare the phyllosphere communities to those in adjacent top soil. Since our results indicate the mānuka phyllosphere microbiome is not recruited stochastically from the surrounding environment, we agree that comparing the phyllosphere microbiomes of mānuka and nearby non-mānuka trees is an obvious target for the next stage of investigation.

15. The authors investigated the differences between geographical as well as elevations, it is unclear to me though if both factors can be separated as the different mānuka host populations were found at different elevations and thus distance and elevation are connected.

**Author response:** We would like to assure the reviewer that there is no autocorrelation between geographic variables (latitude/longitude) and elevation owing to New Zealand’s complex topography. Elevation serves as a proxy for temperature (when coupled with latitude) and geographic distance serves as a test for microbial dispersal (i.e., a distance-decay relationship).

We do acknowledge that host populations separated by larger geographical distances may be more genetically distinct, which may therefore have a confounding influence on the correlation we observed between microbial community dissimilarity and geographical distance (NB: see also the response to comment #17, where we explain why we did not include host genetics in this study). However, if genetic dissimilarities between spatially isolated mānuka populations do have a confounding influence on the structure and composition of phyllosphere microbial communities, it would further contribute to our argument that there is a specific association between the mānuka phyllosphere microbiome and its host.

16. The authors sampled at different times - connected to flowering of the Mānuka, did they analyse the effect of sampling date on communities?

**Author response:** Given that the primary focus of our study (please refer to the response to comment #1 for our revised hypothesis and null hypothesis) was to investigate whether the mānuka phyllosphere microbiome exhibits specific host associations patterns, it was of critical importance that we controlled for known host physiological variations (i.e.,

flowering, see Noe et al., 2019 [43]) by collecting samples from trees in similar stages of flowering. Mānuka populations exhibit a broad (temperature-driven) latitudinal gradient in flowering timing, and our sampling dates were determined accordingly. Our samples do not show any gradient according to sampling date, and we have added sampling date labels to our hierarchical cluster analysis in Fig S7 to highlight this.

***“S7 Fig: Hierarchical clustering analysis of the mānuka phyllosphere microbiome.*** *Branches are coloured according to sample region and sample date. Boxes indicate five cluster groups (k). Branch labels correspond to individual treeID. The length of the branches corresponds with Bray-Curtis dissimilarity between samples.”*

17. I do not understand the paragraph connected to figure 5 or figure 5 itself - this seems overanalysed to me. Phyllosphere communities can be diverse, recent studies indicate that priority effect can have strong influence on the communities - the authors seem to

infer that every plant had the same founder population of bacteria surrounding them. The authors have not included host genetic in this analysis, why? Many others have found that this can impact on the phyllosphere microbiome (e.g. Bodenhausen et al. 2014)

**Author response:** We thank the reviewer for raising these points. Both reviewers commented on the clarity of the paragraph connected to Figure 5 and the figure itself. Please see our response to comment #33 where we corrected these errors.

We absolutely agree that host genetics may have an influence on phyllosphere microbial community structure. However, we reasoned that characterisation of host genetics was not necessary for testing our hypothesis and null hypothesis (please refer to our revised hypotheses in the response to comment #1). In fact, had our results showed that the mānuka phyllosphere microbiome exhibits stochastic recruitment from the surrounding environment, characterisations of host genetics would not have provided useful insight. However, since our findings do indicate that the mānuka phyllosphere microbiome is not recruited stochastically from the surrounding environment, we agree that characterising host genetics is now a worthwhile avenue to explore. We mentioned this in our conclusion when addressing future research opportunities.

Lines 623-630: *“Incorporating host genetic analyses and co-occurring plant species into future analyses will also shed light on the relationship between the mānuka phyllosphere microbiome and host genetics as well as the role of recruitment in community assembly.”*

Our interpretation of the data does not imply or require that each plant be exposed to the same “founder population” of bacteria. Rather, we view the emergence of a prominent core microbiome in the mānuka phyllosphere to be the product of a typical microbial community assembly process where the colonisation success of individual microorganisms is determined by a suite of abiotic and biotic selective forces. The end-product of a core microbiome on all mānuka leaves sampled is consistent with the scenario where specific interactions between the members of the core microbiome and the host (i.e., biotic selection) have a dominant effect in shaping community assembly (i.e., host-specific associations). Note that this process needs not occur within a single colonisation event—community succession is an integral part of assembly. The Bodenhausen et al. study has limited direct relevance for how we may interpret our findings since their experimental system was composed of an artificial community inoculated onto gnotobiotic plants. Nevertheless, our interpretation of the data is compatible with the overall finding of the Bodenhausen et al. study, which is that the phyllosphere microbiome can show host-specific associations.

18. The PICRUSt analysis seems another step of over analysing the data - what is the purpose of this within the study as a whole? If anything, it would have been useful to try and identify genes that may be responsible for changes in the amount of active compounds in the Mānuka honey to attempt the provide evidence for the authors initial hypothesis. Here it is merely used to contrast leaf from soil - which has been achieved by the 16S diversity already and does not add new information.

**Author response:** We agree with the reviewer that the PICRUSt analysis did not provide particularly novel insight, and we absolutely recognise the caution required in interpreting this type of predicted microbial functions. However, given that the mānuka phyllosphere core microbiome contains bacterial phyla commonly found in other phyllosphere microbiomes (see our response to comment #13, where we compare and contrast the mānuka phyllosphere microbiome with comparable studies), we felt that PICRUSt predictions may provide some useful context, particularly in further contrasting the difference between phyllosphere and soil microbiomes. To avoid giving PICRUSt data more credibility than is warranted, we have moved Figure 6 to Supplementary Material as Figure S10.

19. Discussion line 410: I agree with the claim made, but the references used seem off - there is studies that show that phyllosphere bacteria impact on the host directly e.g. Vogel et al. 2016 (DOI: 10.1111/nph.14036).

**Author response:** We thank the reviewer for pointing this and we have updated the citations associated with this sentence which now reads as follows:

Lines 474-475: *“The phyllosphere microbiome is increasingly recognised as an influential component of host plant biology [4-6].”*

4. Vogel C, Bodenhausen N, Gruissem W, Vorholt JA. The Arabidopsis leaf transcriptome reveals distinct but also overlapping responses to colonization by phyllosphere commensals and pathogen infection with impact on plant health. New Phytologist. 2016;212(1):192-207.

5. Cid FP, Maruyama F, Murase K, Graether SP, Larama G, Bravo LA, et al. Draft genome sequences of bacteria isolated from the Deschampsia antarctica phyllosphere. Extremophiles. 2018;22(3):537-52.

6. Kumaravel S, Thankappan S, Raghupathi S, Uthandi S. Draft genome sequence of plant growth-promoting and drought-tolerant Bacillus altitudinis FD48, isolated from rice phylloplane. Genome Announc. 2018;6(9):e00019-18.

20. The authors claim that they are the first to identify a core microbiome despite that others have performed similar studies with similar tools. Does that mean that Mānuka is different compared to many other plants and that the results here cannot be generalised over many other plant species? I would think so - but I do not read in the provided manuscript.

**Author response:** Our claim is based on a stringent and microbiologically relevant definition of a phyllosphere core microbiome. Please see the responses to comments #5 and #10, where we highlight the inconsistencies in how both the core microbiome concept and phyllosphere have been defined across scientific literature, respectively, and why these inconsistencies create challenges for microbial ecology research.

As for the generalisability of our findings—the presence of such a prominent phyllosphere core microbiome may indeed be a rare phenomenon (although not likely unique to mānuka). Please also see the response to comment #13, where we discuss the

uniqueness of the mānuka phyllosphere microbiome compared to the phyllosphere of other plants. Going forward, we suggest that investigation of other phyllosphere microbiomes should be conducted *sensu stricto* as per our definition in order to produce directly comparable data, and this is potentially the most generalisable message from our findings. We have made changes within the conclusion to address these points:

Lines 631-640: *“Understanding potential roles of the mānuka phyllosphere microbiome in the physiology of the nationally and culturally treasured indigenous plant will be of wide scientific and general interest. Our findings also illustrate the usefulness of the mānuka phyllosphere microbiome for expanding our knowledge of specific host-microbe associations in the phyllosphere and support the notion that investigation of phyllosphere microbiomes should be conducted as per our sensu stricto definitions of the phyllosphere and core microbiome to ensure relevance and generalisability of findings.”*

21. Line 429 references 80 and 81 do not fit here.

**Author response:** We thank the reviewer for pointing this out. These references have been removed, and the corresponding text within the discussion has been changed in response to comment #13, so that we now compare and contrast our results with other phyllosphere studies. The corresponding section of the discussion now reads as follows:

Lines 507-517: *“The apparent host-specific association in the mānuka phyllosphere microbiome supports previous studies that identified species-specific communities in the phyllosphere of co-occurring plant species [24]. Genotype-dependent characteristics of leaf morphology and chemistry have been suggested to drive such host-specific patterns [28]. Notably, medicinal plants have previously been reported to harbour highly specialised microbial communities due to their unique and structurally divergent secondary metabolites [67]. The unique components of mānuka’s antimicrobial essential oils may therefore play a role in the selection and maintenance of specific bacterial lineages in its phyllosphere across large geographical ranges and variable environments [68].”*

24. Laforest-Lapointe I, Messier C, Kembel SW. Host species identity, site and time drive temperate tree phyllosphere bacterial community structure. Microbiome. 2016;4:10. doi: 10.1186/s40168-016-0174-1. PubMed PMID: WOS:000378621200001.

28. Kembel SW, O’Connor TK, Arnold HK, Hubbell SP, Wright SJ, Green JL. Relationships between phyllosphere bacterial communities and plant functional traits in a neotropical forest. Proceedings of the National Academy of Sciences. 2014;111(38):13715-20.

67. Köberl M, Schmidt R, Ramadan EM, Bauer R, Berg G. The microbiome of medicinal plants: diversity and importance for plant growth, quality and health. Frontiers in microbiology. 2013;4:400-. doi: 10.3389/fmicb.2013.00400. PubMed PMID: 24391634.

68. Douglas MH, van Klink JW, Smallfield BM, Perry NB, Anderson RE, Johnstone P, et al. Essential oils from New Zealand manuka: triketone and

other chemotypes of Leptospermum scoparium. Phytochemistry. 2004;65(9):1255-64. doi: 10.1016/j.phytochem.2004.03.019.

22. Line 432 since the sentence should relate to the phyllosphere the reference 83 cannot be used as it studies endophytic microbes

**Author response:** We thank the reviewer for pointing out this mistake, and we have removed this reference. The text now reads as follows:

Lines 511-514: “*Notably, medicinal plants have previously been reported to harbour highly specialised microbial communities due to their unique and structurally divergent secondary metabolites [67].”*

67. Köberl M, Schmidt R, Ramadan EM, Bauer R, Berg G. The microbiome of medicinal plants: diversity and importance for plant growth, quality and health. Frontiers in microbiology. 2013;4:400-. doi: 10.3389/fmicb.2013.00400. PubMed PMID: 24391634.

23. Line 442-444 Sphingomonads in the study did not actively antagonise Pseudomonas - in fact refer to the Vogel paper further up - apparently Sphingomonas Fr1 activate plant immune responses which allowed for a faster response of the plant towards the pathogen, also Sphingomonads can also be plant pathogens Buonaurio et al. 2002 (DOI: 10.1099/ijs.0.02063-0), so this claim might not hold.

**Author response:** We thank the reviewer for pointing this out, and we have made amendments to the text to make the reference more appropriate.

Lines 527-529: *“For example, Sphingomonas has been shown to enhance the host plant immune response towards pathogenic strains of Pseudomonas [69].”*

69. Innerebner G, Knief C, Vorholt JA. Protection of Arabidopsis thaliana against leaf-pathogenic Pseudomonas syringae by Sphingomonas strains in a controlled model system. Appl Environ Microbiol. 2011;77(10):3202-10. Epub 2011/03/23. doi: 10.1128/aem.00133-11. PubMed PMID: 21421777; PubMed Central PMCID: PMCPMC3126462.

24. The cross-contamination paragraph is odd and uncommon to see in such a manuscript - it is better suited in a supplement or in a protocol paper. I suggest to delete it from the main manuscript.

**Author response:** We recognise that eliminating cross contamination seems like a methodological detail; however, cross contamination is an entirely reasonable alternative explanation for our findings (i.e., a consistent group of contaminants as opposed to a core microbiome across all samples). We therefore believe that this issue requires specific attention in the discussion. We have added a sentence to the beginning of the paragraph to make our rationale for including this paragraph clearer.

Lines 581-583: “*Our study was strengthened by aspects of our methodological design that allowed us to essentially exclude cross-contamination as an alternative explanation for the presence of a ubiquitous core microbiome.”*

25. After reading the article, I want to learn more about the core-microbiome of mānuka - but the authors are not addressing this question - what organisms can we find across Mānuka, are they common on other plants, what are their likely properties, do they have plant growth promoting properties, are they plant pathogens in other systems, or antagonists etc.?

**Author response**: We thank the reviewer for this insightful suggestion. We have made changes to the conclusion when addressing future research opportunities to make this point:

Lines 616-630: “*Although the mānuka core phyllosphere microbiome was prominent, their functions cannot be reliably inferred from 16S rRNA gene PCR amplicon sequences. The immediate next step should be to investigate their functional potential and their role in mediating the physiological properties of mānuka using a combination of shotgun metagenomic sequencing and cultivation approaches. Metagenomics will also reveal the diversity and abundance of micro-eukaryotes such as fungi and protists, which are increasingly recognised as potential determinants of plant-microbe interactions and host fitness. Incorporating host genetic analyses and co-occurring plant species into future analyses will also shed light on the relationship between the mānuka phyllosphere microbiome and host genetics as well as the role of recruitment in community assembly.*”

**Comments from Reviewer 2**

In this manuscript, the authors measured the leaf and soil microbial communities across five distinct populations of Leptospermum scoparium (total of 29 individual tree sampled, ~ 6 trees per site, three branches per tree), an indigenous tree species to NZ. The authors hypothesize that the phyllosphere microbiota plays a role in mediating host physiology and thus exhibits specific host association. They identify a habitat specific and abundant leaf core microbiome present across all samples and claim that this result supports their hypothesis that phyllosphere microorganisms exhibit host association and potentially mediate physiological traits.

26. This manuscript discusses the relevance and the definition of a core microbiome, which provides pertinent information to the field of host-microorganism interactions. However, the fact that the authors excluded voluntarily the endophytic communities by modifying their protocol make me doubt the accuracy of their claim to have identified microorganisms that mediate plant physiological traits as endophytes are recognized to be key organisms interacting with plant local and systemic immune system. The exploration of biogeography patterns in leaf bacterial communities for a single tree species contributes relevant information to the field.

**Author response:** We would like to thank the reviewer for their insightful comments and helpful feedback. Firstly, we would like to direct the reviewer’s attention to a number of important revisions made in response to comments made by reviewer 1. We feel the most important of these revisions is the clarification of our two-part hypothesis that underpins our broader research: 1) that the mānuka phyllosphere microbiome plays a role in mediating host physiology, and therefore 2) the mānuka phyllosphere microbiome exhibits specific host association. We acknowledge that only the latter was testable within the context of the analyses included in our manuscript, and we have clarified both the testable hypothesis and its corresponding null hypothesis in the revised manuscript (please see our response to comment #1 for specific changes to the manuscript).

Our decision to focus exclusively on the leaf surface epiphytes was driven by recent evidence that suggests the phyllosphere microbiome has an underappreciated role in the functioning of their host plants and wider terrestrial ecosystem (please see the response to comment #2 where we explain the underappreciated role of the phyllosphere microbiome in more detail). Given the potential of the phyllosphere microbiome to influence various aspects of host plant physiology and its unevaluated role in mānuka, we therefore applied the core microbiome conceptual framework to investigate whether the mānuka phyllosphere exhibits host association patterns congruent with that of a phyllosphere microbiome that may mediate host physiology (please refer to our revised hypotheses in the response to comment #1). As different plant microhabitats, such as the endosphere and phyllosphere, harbour taxonomically and functionally distinct microbial communities, we do not believe excluding the endophytic compartment invalidates the patterns of specific host-association observed in the phyllosphere, nor its potential to mediate aspects of host physiology.

27. Introduction: The authors should discuss or at least acknowledge the limits of describing only leaf bacterial communities when micro-eukaryotes such as fungi and protists but also phages are increasingly recognized to play a key role as determinants of plant-microbe interactions and host fitness.

**Author response:** We thank the reviewer for this suggestion. As epiphytic bacteria are typically the most abundant coloniser of the phyllosphere, we decided to focus our first investigation of the mānuka phyllosphere microbiome on bacteria. We do acknowledge that epiphytic fungi can play important roles in the phyllosphere and will endeavour to include them in future research. We also plan to conduct shotgun metagenomic sequencing of the mānuka phyllosphere microbiome, which will shed light on microeukaryotes and phages. We have made changes within the introduction, which now acknowledges the full diversity of the phyllosphere microbiome:

Lines 41-51: “*Plants harbour distinct and dynamic environments colonised by complex microbial communities known as host microbiomes [1]. Evidence has shown that interactions between plants and their associated microorganisms in many of these microbiomes play a pivotal role in host plant health, function, and evolution [2]. The leaf surface, or phyllosphere, harbours a microbiome comprising diverse communities of bacteria, fungi, algae, archaea, and viruses [3]. Interactions between the host plant and phyllosphere bacteria have the potential to drive various aspects of host plant*

*physiology [4-6]. However, our knowledge of these bacterial associations in the phyllosphere remains relatively modest, and there is a need to advance fundamental knowledge of phyllosphere microbiome dynamics [7].”*

Lines 124-130: “*Microorganisms have been studied in the mānuka rhizosphere and endosphere [46-48]. Although existing studies have primarily focussed on fungi, a recent study provided the first investigation of endophytic bacterial communities from three geographically and environmentally distinct mānuka populations using fingerprinting techniques and revealed tissue-specific core endomicrobiomes [49]. However, the role of microorganisms in driving DHA variation remain unknown, and a characterisation of the mānuka phyllosphere microbiome has not been conducted.”*

We have also made change within our conclusion to acknowledge the value of incorporating micro-eukaryotes such as fungi and protists in future research:

Lines 616-630: *“Although the mānuka core phyllosphere microbiome was prominent, their functions cannot be reliably inferred from 16S rRNA gene PCR amplicon sequences. The immediate next step should be to investigate their functional potential and their role in mediating the physiological properties of mānuka using a combination of shotgun metagenomic sequencing and cultivation approaches. Metagenomics will also reveal the diversity and abundance of micro-eukaryotes such as fungi and protists, which are increasingly recognised as potential determinants of plant-microbe interactions and host fitness. Incorporating host genetic analyses and co-occurring plant species into future analyses will also shed light on the relationship between the mānuka phyllosphere microbiome and host genetics as well as the role of recruitment in community assembly.”*

28. Methods: Why use surface soil and not root associated microorganisms?

**Author response:** Previous studies have suggested the phyllosphere microbiome are recruited from free-living microbial communities in surrounding soil. Therefore, to test our null hypothesis that the mānuka phyllosphere microbiome is recruited stochastically from the surrounding environment (please see the response to comment #1 for our revised hypotheses), we chose to compare the phyllosphere communities to those in soil (NB: please also see the response to comment #14 where we explain why we used surface soil rather than other tree species). The assembly and composition of the rhizosphere microbiome is determined by a suite of abiotic and biotic selective forces that will be specific to the rhizosphere, and it therefore would not have provided a suitable comparison to test our null hypothesis. However, we do agree that comparing the phyllosphere microbiome to other microbial communities associated with mānuka (such as the rhizosphere) is an interesting avenue for future research.

29. Figure S1. Would it be possible to add more information to this figure by overlaying changes in topography, or forest/land type, mean temperature, etc.

**Author response:** We thank the reviewer for this suggestion. Overlaying changes in topography is, however, difficult to illustrate at the scale represented in Figure S1 due to New Zealand’s complex topography. We have also refrained from mapping our sample

locations at local scales to preserve their anonymity (a condition of access approval for some sites). We have, however, replaced the map in Figure S1 so that it now shows regional boundaries of the North Island, which are highly depictive of different climatic zones. Elevation, monthly rainfall (mm), and sun (hrs) for each site have also been included to provide the reader with additional information. The figure legend reads as follows:

***“S1 Fig: Location of Leptospermum scoparium (mānuka) populations in the North Island of New Zealand.*** *Borders represent regional boundaries. Total rain (mm) and total sun (mm) data retrieved from the National Climate Database (NIWA) for the month prior to sampling. Map made with Natural Earth.”*

30. Figure 1. Panel A. It would be relevant to have a third panel equivalent to panel A where relative abundance is collapsed per site to present the mean relative abundance of core OTUs because there seems to be site specific trends. Panel B can be rescaled to provide better resolution by having 0.5% as the maximum on the y axis. As of now we barely see anything.

**Author response:** We thank the reviewer for these specific and useful suggestions. We now include a third panel in Figure 1, and panel B has been rescaled. Figure 1 legend now reads as follows:

Line 328-330: “***Fig 1. Relative abundance of core phyllosphere taxa in the mānuka phyllosphere (A &C) and associated soil communities (B).*** *Relative abundance is averaged per sample tree (A) and site (C).”*

31. Lines 279-280: How much variation in BC dissimilarity did habitat (leaf vs. soil) explain?

**Author response:** Habitat (leaf vs. soil) explained 41% of the total variation in Bray-Curtis dissimilarity between phyllosphere and soil samples. We apologise for omitting this information and have now done so:

Lines 341-343: *“At the OTU level, phyllosphere and soil community compositions were significantly different, with habitat explaining 41% of the variation in Bray-Curtis dissimilarities (****Error! Reference source not found.*** *& PERMANOVA on Bray-Curtis dissimilarities, P< 0.001).”*

32. Lines 284-285: It is intriguing that no soil core microbiome was detected, what could explain this?

**Author response:** We thank the reviewer for raising this question. Mānuka is renowned for its adaptability to a wide range of environments, including marginal land, and our sample sites were indeed very diverse in terms of microclimate and geography. Each of our five sites exhibited differences in latitude/longitude and elevation, which in turn drives differences in precipitation and temperature, among other variables. It is therefore not surprising a soil core microbiome was not observed, and this finding further highlights the significance of identifying a core microbiome in the phyllosphere.

33. Lines 368-370: I don’t understand what it means to “represent independently and collectively 42% of the variation in community structure”. Also, are the numbers in Fig. 5 representing %? Is so this should be indicated in the figure or stated in the legend.

**Author response:** We thank the reviewer for pointing out this error. We have clarified this sentence and included % in the corresponding figure legend. The revised text reads as follows:

Lines 431-435: *“Variation partitioning was used to further resolve the relative contributions of environmental variables, spatial factors, and host traits on the total phyllosphere community structure. In total, 42% of the phyllosphere community variation was explained by these three groups of explanatory variables (Fig 5).”*

Lines 448-455: *“****Fig 5: The proportion (%) of mānuka phyllosphere microbiome variation explained by environmental, spatial, and host tree variables.*** *Results of partial regression analysis show the proportion of variation attributable to each individual and combination of explanatory variables. Environmental variables (ENV) represent the night-day temperature differential, night temperature, monthly precipitation, and monthly cloud cover. Spatial variable (DIST) represents the straight-line distance between sample coordinates. Host tree variables (HOST) represent tree height and tree diameter.”*

34. Line 448 (and other places): I would refrain from using the word “patterning” as this term is rarely used (to not say never). Please use “patterns” or “dynamics” to use literature-relevant terminology.

**Author response:** The word “patterning” has been replaced with the word “patterns”, and the revised texts read as follows within the discussion and conclusion, respectively:

Lines 538-548: “*Besides harbouring a core microbiome, the mānuka phyllosphere exhibited biogeographical patterns that appeared to be shaped by both deterministic and neutral processes [72, 73]. Biogeographical patterns have been identified in the phyllosphere of other tree species [21, 74]. However, few studies have examined the biogeographical patterns of different microbial populations in the phyllosphere (e.g., rare versus abundant and core versus non-core taxa) as in other environments [8, 26, 75]. Our results revealed that biogeographical patterns were strongest in non-core taxa, exhibiting a non-random distribution across space and environmental gradients that contrasted with the cosmopolitan distribution of the relatively dominant core microbiome.”*

Lines 604-615: *“Our findings demonstrate that Leptospermum scoparium (mānuka) possesses a dominant core phyllosphere microbiome that is persistent across geographically distant populations. Core phyllosphere taxa represented a large proportion of the total phyllosphere community yet were rare or entirely absent in surrounding soils, providing support for hypothesised specific host association and potentiating the role of host selection of functionally important microorganisms. Strong biogeographical patterns were also identified in the mānuka phyllosphere, indicating that spatially structured environmental gradients may play a role in shaping the composition of non-core microbiota. Together, these findings illustrate that the mānuka phyllosphere microbiome exhibits complex biogeography that likely resulted from divergent processes driving the assembly of core and non-core microorganisms.”*

35. Lines 480-483: This is shocking to me. Excluding the leaf endophytes might have dramatic consequences as they are expected to be agents interacting directly with the plant host. Please discuss the relevance of excluding endophytes and the limits this imposes on your study.

**Author response:** We would like to direct the reviewer to our response to comment #26, where we have explained our rationale for characterising the bacterial leaf epiphytes exclusively and why we respectfully maintain excluding endophytes does not invalidate the data presented by this study.

36. Cross-contamination: It seems to me that the section on cross-contamination rather belong in the methods.

**Author response:** Please see the response to comment #24, where we have clarified our rationale for including the paragraph on cross-contamination in the discussion.

37. Lines 492-495: This also has consequences for the literature where most papers have suggested that soil is a reservoir for leaf microorganisms. Please compare your results with corresponding references that have found opposite results to yours and cite these references.

**Author response:** We thank the reviewer for making this point. Indeed, previous studies have suggested soil is a reservoir for phyllosphere microorganisms. These suggestions provided the foundation for our null hypothesis that the mānuka phyllosphere microbiome is recruited stochastically from surrounding soil (please refer to our revised hypotheses in the response to comment #1). Contrary to the suggestions made by these previous studies, our results provide evidence in support of biotic selection having a dominant role in shaping community assembly of the mānuka phyllosphere microbiome (i.e., host-specific associations). We have added a short discussion on how our results compare and contrast with these studies in our discussion:

Lines 561-579: ***“****Significant differences in community composition were observed between the host microbiome in the phyllosphere and free-living communities in the surrounding soil. As expected, soil bacterial communities were more diverse than those in the phyllosphere. Given the environmental heterogeneity of the surrounding environments in which we sampled, the collection of neutral and deterministic forces shaping the soil communities were likely very diverse. Notably, taxa that were abundant in the phyllosphere, including the core microbiome, were essentially absent in surrounding soil. This finding contrasts with previous studies that report a large proportion of abundant phyllosphere taxa are also present in soil [25, 79]. Although the major reservoir of the mānuka phyllosphere microbiome cannot be directly inferred from our analysis, if phyllosphere bacterial communities were assembled stochastically from the surrounding environment, the diversity of the surrounding soils would have been expected to drive large regional differences as observed in other plant species, such as the grape vine [25]. Instead, the convergence consistently observed in the phyllosphere microbiome independent of the surrounding environment suggests that soil has limited influence on the community structure of the mānuka phyllosphere microbiome.”*

38. Lines 500-503: It seems to me that the immediate next step is rather to confirm if this leaf core microbiome is truly playing a role for its host fitness and phenotype.

**Author response:** We thank the reviewer for this insightful suggestion and would like to note that this was also a comment made by reviewer 1 (see comment #25). We agree with this suggestion, and we have included this in our discussion when addressing future research opportunities:

Lines 616-630: “*Although the mānuka core phyllosphere microbiome was prominent, their functions cannot be reliably inferred from 16S rRNA gene PCR amplicon sequences. The immediate next step should be to investigate their functional potential and their role in mediating the physiological properties of mānuka using a combination of shotgun metagenomic sequencing and cultivation approaches. Metagenomics will also reveal the diversity and abundance of micro-eukaryotes such as fungi and protists, which are increasingly recognised as potential determinants of plant-microbe interactions and host fitness. Incorporating host genetic analyses and co-occurring plant species into future analyses will also shed light on the relationship between the mānuka phyllosphere microbiome and host genetics as well as the role of recruitment in community assembly.*”

39. Figure 3. I find it strange to see that hierarchical cluster was able to perfectly separate samples per site provenance based on bacterial community structure. I would expect that stochasticity would at least have mixed some samples into different site provenance. Could you provide an NMDS/PCoA showing how clearly these five clusters separate based on community structure?

**Author response:** We thank the reviewer for pointing this out. We did not transform the relative abundances, which means that low-abundance OTUs are de-weighted in the clustering analysis. We do agree, however, that an nMDS may more accurately display the clustering of phyllosphere communities belonging to different site provenances. We have replaced Figure 3 with an nMDS and moved the hierarchical cluster into the Supplementary Material as Figure S7.

Lines 372-377: *“****Fig 3: Nonmetric multidimensional scaling (NMDS) showing the community structure of the mānuka phyllosphere microbiome across sites.*** *Distance based on Bray-Curtis dissimilarity. The stress value of the NMDS plot is 0.15.”*
