# Supplementary material for: A core phyllosphere microbiome exists across distant populations of a tree species indigenous to New Zealand
Source: PLoS One. 2020 Aug 13;15(8):e0237079. doi: 10.1371/journal.pone.0237079 (PMC7425925; doi:10.1371/journal.pone.0237079)
Supplement: Attachment — Submitted filename: Response to Reviewers.docx [file pone.0237079.s024.docx]

# Comments from the Editor

For this second round of reviews, only one of the original reviewers has agreed to proceed with the revision. As you can see, this reviewer still has some important concerns. In the absence of another reviewer I have stepped in and I have some additional comments:

**Author response:** We would like to thank you for the time taken to provide these additional comments.

1. I think for the core taxa analysis a slightly more sophisticated approach could be taken. The different samples are not rarefied and you completely discount relative abundance. The combination of both of these factors could distort your selection criteria. I would suggest to (a) rarefy your samples to a uniform size for this analysis and (b) plot relative abundance against site prevalence (in how many sites is this OTU detected). you can draw lines to demarkate your selection criteria, but it will give the reader an idea of how many OTUs were very close but out of the bounds of your criteria.

**Author response:** We agree that there are many different approaches that can be utilised to define core microbiota (as recently reviewed by Shade and Stopnisek, 2019 [69], doi: 10.1016/j.mib.2019.09.008). Owing to the explicit spatial hierarchies in our sampling design and the high environmental heterogeneity between sampled populations, we prioritised taxon occupancy as our criterion for defining core taxa over abundance. This choice was also supported by the knowledge that many microbial strains with functional implications for plants are often rare, and abundance-defined cores may therefore miss important members that are not dominant in the community. However, we do acknowledge that a 100% occupancy threshold is a highly conservative approach and will bias toward more abundant taxa. Nevertheless, this conservative approach will also be less likely to include false positive taxa.

(a)

Sequence reads were rarefied on a per-sample basis using rarefy_even_depth implemented in the phyloseq package with rarefaction set to the 5th percentile of per sample sequence reads (3,979). Five samples were excluded from rarefaction due to low sequence reads but still included in the core microbiome analysis. Using 100% occupancy as the selection criterion, we identified a core microbiome of 10 OTUs in the rarefied dataset as we did in the unrarefied dataset. Given that contrasting views exist regarding the necessity and validity of rarefaction (McMurdie and Holmes 2014, doi:10.1371/journal.pcbi.1003531), and identical results were obtained using both rarefied and non-rarefied data, we have kept the non-rarefied data for this analysis and downstream analyses within the manuscript.

(b)

We now provide an abundance vs. occupancy plot using both rarefied and non-rarefied data, which can be found as panel D in Fig 1. This figure provides additional insight regarding the relative abundance and prevalence of OTUs identified in the phyllosphere, such as those identified as part of the core microbiome and other OTUs that were very close but outside the bounds of our selection criteria. Importantly, this additional figure allows the readers to conduct their own evaluation of our core microbiome selection.

These changes now read as follows:

In Material and Methods:

Lines 237-240:

*“A rarefied dataset was generated by rarefying reads on a per-sample basis to the 5th percentile of per-sample sequence reads (3,979) using rarefy_even_depth implemented in the phyloseq package [58]. Five samples were excluded from rarefaction due to low numbers of sequence reads.”*

Lines 241-244:

## “Statistics and data analysis

*Data analyses and visualisation were performed using non-rarefied data in R version*

*3.4.3 [59], with the packages DESeq2 [60], ggplot2 [61], phyloseq [58], and vegan [62].”*

Lines 280-290:

## “The core microbiome

*The core mānuka phyllosphere microbiome was identified by plotting OTU abundance and occupancy. An OTU presence in all 89 phyllosphere samples (100% occupancy) was chosen as a highly conservative representation of the core microbiome. In selecting 100% occupancy as the stringent core microbiome criteria, we minimised the likelihood of these taxa representing transient taxa [69]. The core phyllosphere microbiome at 100% occupancy was annotated in both rarefied and non-rarefied datasets.”*

In Results:

Lines 326-344:

## “Mānuka phyllosphere core microbiome

*“Of the 1,384 bacterial OTUs identified in the total phyllosphere community, 10 OTUs were identified in all phyllosphere samples (100% occupancy) and therefore defined as members of the core phyllosphere microbiome (Fig 1D). These 10 core taxa were affiliated with four phyla and five classes: Alphaproteobacteria (Rhizobiales [29.3% of all reads], Sphingomonadales [4.9%]), Bacteroidetes (Cytophagia [2.5%]), Verrucomicrobia (Spartobacteria [2.4%]), and Acidobacteria (Acidobacteriia [1.0%]) (Fig 1A and 1C). All 10 core OTUs were relatively abundant and collectively represented 40.7% of all reads (S7 Table). The most abundant of these 10 core taxa was an unclassified member of the Alphaproteobacteria class in the order Rhizobiales (OTU2), which accounted for 11.6±6.8% of reads in all phyllosphere samples. At the genus level, the core taxa are affiliated with Hymenobacter, Sphingomonas, Terriglobus, Spartobacteria genera incertae sedis, and unclassified genus within the Rhizobiales order. BLASTn analysis of the 10 core OTUs revealed the closest relatives were isolates from diverse environments such as freshwater, soil, and plant material (S10 Fig).”*

Lines 346-352:

***“Fig 1: Relative abundance of core phyllosphere taxa in the mānuka phyllosphere (A, C) and associated soil communities (B).*** *Relative abundance is averaged per tree (A) and site (C). An abundance-occupancy distribution was used to identify core phyllosphere taxa in non-rarefied (green) and rarefied (purple) datasets (D). Each point represents a taxon plotted by its mean log10 relative abundance and occupancy. Taxa (pink) with an occupancy of 1 (i.e., detected in all 89 phyllosphere samples) were considered members of the core microbiome. Panels A – C are colour- coded by OTU.”*

2. In line 303 you mention you used blastn to identify the taxonomy of your strains. It is not clear to me why you needed to do this considering the taxonomy was already assigned using RDP, which is a superior way to assign taxonomy to 16S reads. If you would like to explore the taxonomic neighborhood of these taxa, I would build a phylogenetic tree out of close RDP or blast hits and place your sequenceds within it.

**Author response:** Thank you for this helpful suggestion. Indeed, we used blastn to explore the taxonomic neighbourhood and isolation sources of the closest relatives to the core taxa. As per your suggestion, we have now included a phylogenetic tree out of the closest blastn sequences and placed the sequences of the 10 core taxa within it. This figure can be found in the supplementary material as S10 Fig and has replaced S8 Table.

In Material and Methods the text now reads:

Lines 272-279:

*“The nucleotide Basic Local Alignment Search Tool (BLASTn) algorithm was used to identify isolates with the closest sequence identity to core taxa. Core and BLASTn sequences with the highest nucleotide identity (%) were aligned with ClustalW. A phylogenetic tree was constructed in MEGA X [67] using Maximum Likelihood method and Tamura-Nei model [68].”*

In Results the text now reads: Lines 340-344*:*

*“BLASTn analysis of the 10 core OTUs revealed the closest relatives were isolates*

*from diverse environments such as freshwater, soil, and plant material (S10 Fig).”*

Lines 1294-1299*:*

*“S10 Fig: Phylogenetic maximum-likelihood analysis based on 16S rRNA gene PCR amplicon sequences of core phyllosphere OTUs and bacterial isolates with the closest sequence identity. Isolates were identified using BLASTn; the accession number and isolation source of each isolate is provided. Sequences were aligned with ClustalW. Bootstrap probabilities based on 1000 replications are shown. Bar: 0.05 nucleotide substitutions rate (Knuc) units.”*

3. Since you've sequenced all of these soil samples, I think it would be more interesting if you ran a model to compare soil vs phyllosphere for each OTU rather than simply reporting on the overlap. That way you can also find our whether your core OTUs are indeed shoot enriched in a quantitate way.

**Author response:** We thank the editor for making this comment and agree with the suggestion. We now include an analysis of the differential abundance of the OTUs that are shared by both phyllosphere and soil communities. This analysis was performed using DESeq2. A full list of OTUs with significant differential abundances, defined by DeSeq2 log-fold difference with an adjusted p value of ≤ 0.01, is presented in S8 Table. Using this

analysis, we were able to identify that the 9 core OTUs detected in very low relative abundances in some soil samples (1 core OTU was absent from all soil samples) were indeed significantly enriched in the phyllosphere.

In Materials and Methods, the text now reads: Lines 241-244:

## “Statistics and data analysis

*Data analyses and visualisation were performed using non-rarefied data in R version*

*3.4.3 [59], with the packages DESeq2 [60], ggplot2 [61], phyloseq [58], and vegan [62].”*

Lines 277-279*:*

*“The R package ‘DESeq2’ was used to identify OTUs that are differentially abundant across phyllosphere and soil communities, and estimates of log-fold changes were obtained [60].”*

In Results, the text now reads: Lines 366-379:

*“At the OTU level, phyllosphere and soil community compositions were significantly*

*different, with habitat explaining 41% of the variation in Bray-Curtis dissimilarities (Fig 2A & PERMANOVA on Bray-Curtis dissimilarities, P< 0.001). Notably, a soil core microbiome was not detected. Overall, 599 OTUs were shared by at least one soil and leaf sample, rendering 783 and 6,306 OTUs habitat-specific to the leaf surface and surrounding soil environment, respectively. Of the 599 OTUs shared by soil and phyllosphere communities, 256 exhibited significant differential abundances between habitat types; 152 and 104 were significantly abundant in soil and phyllosphere samples, respectively (Fig 2B & S8 Table). Nine core phyllosphere taxa were detected in at least one soil sample (S7 Table). However, core phyllosphere taxa were present at very low relative abundances in soil and exhibited significant enrichment in the phyllosphere (Fig 1B & S11 Fig).”*

Lines 381-390:

***“Fig 2. Differential abundance of taxa in phyllosphere and associated soil communities.*** *(A) Heatmap depicts the differential relative abundance of OTUs in each environment. Only OTUs with mean relative abundance greater than 1 x 10^-3^ are included. (B) OTUs present in both phyllosphere and soil communities that exhibit significant differential abundances. Positive log2 fold change values represent OTUs with increased abundance in the phyllosphere relative to soil (n=104) and those with negative log2 fold change values represent OTUs with increased abundance in soil relative to the phyllosphere (n=152). A full list of OTUs with significant differential abundances, defined by DeSeq2 log-fold difference with an adjusted p value of ≤ 0.01, is presented in S8 Table.”*

Lines 1300-1304:

*“S11 Fig: OTUs with significantly increased abundance in the phyllosphere relative to soil (n=104). Estimates of log2 fold changes were obtained using DESeq2. Colour depicts members of the core phyllosphere microbiome. A full list of OTUs with significant differential abundances, defined by a DeSeq2 log-fold difference with an adjusted p value of ≤ 0.01, is presented in S8 Table.”*

*Lines 1326-1327:*

*“S8 Table: Full list of OTUs with significant differential abundances, defined by DeSeq2 log-fold difference with an adjusted p value of ≤ 0.01.*

# Comments from Reviewer 1

4. The authors have added a lot of text and edited their manuscript heavily - I am surprised to see a strong reluctance to remove the strong focus on DHA from the manuscript even though both reviewers are concerned about this. I would consider the manuscript suitable for publication without it.

They also added some additional introduction and discussion that I find unnecessary and rather irritating.

**Author response:** We would like to thank the reviewer once again for their comments and the time taken to provide these insightful critiques and suggestions. The cause of DHA heterogeneity in mānuka nectar is a current scientific enigma, and we agree with the reviewer that this study does not directly contribute to the topic of DHA (for specific comments please see #11-14 below). It was not our intention to imply that this study delivers any specific information regarding DHA heterogeneity, and we apologise for giving the reviewer this incorrect impression. We agree with the reviewer (comment #11) that the knowledge built here can in the future be useful to explore reasons for differing DHA concentrations. To further clarify this, we have made specific changes in response to comments #11-14 below, where the reviewer has outlined specific text within our introduction that required attention. We have made these recommended changes within each of these comments. We hope the changes made in response to these comments alleviate any remaining concerns the reviewer may have regarding DHA.

5. The conclusion is too long - part of it should be moved into the discussion i.e. Lines 616 - 630 should not be part of a conclusion

**Author response:** As requested by Reviewer 1 and 2 in the first review, we included additional text to acknowledge the limitation of describing only bacterial communities (review1, comment #27) and the next steps for future research (review 1, comment #25 and #38). However, we agree with the reviewer that this text is better suited to the discussion. We have resolved this by removing lines 616-630 from the Conclusion and incorporating this into the Discussion. The text now reads as follows:

Lines 599-633:

## “Future directions

*Our study was strengthened by aspects of our methodological design that allowed us to essentially exclude cross-contamination as an alternative explanation for the presence of a ubiquitous core microbiome. Specifically, soil samples were processed simultaneously with leaf samples to mitigate the risk of cross-contamination during sample collection or processing, and fusion primers were used to prepare 16S rRNA gene PCR amplicons in a one-step protocol to eliminate the risk of cross-contamination by aerosolised PCR products [47-51]. The immediate next step should be to investigate the functional potential and role of the core phyllosphere microbiome in potentially mediating the physiological properties of mānuka using a combination of shotgun metagenomic sequencing and cultivation approaches. Metagenomics will also reveal the diversity and abundance of micro-eukaryotes such as fungi and protists, which are increasingly recognised as potential determinants of plant-microbe interactions and host fitness [8]. Incorporating host genetic analyses and co-occurring plant species into future analyses will also shed light on the relationship between the mānuka phyllosphere microbiome and host genetics as well as the role of recruitment in community assembly.”*

6. Looking into the use of just a few of references that were used here as mentioned below, I still think that the authors will have to do their due diligence and carefully check the context and content of all the references that they are using!

I hope that the authors are less reluctant to take advice this time. The data and biogeography aspect of this study is certainly worth publishing.

**Author response:** We would like to thank the reviewer for their comment and apologise for errors in our referencing. We have made changes to the references specifically mentioned in comments #8, #9, #10, and #18 below. We have also carefully considered all other citations.

*Specific comments*

7. Line 24 and 25. “association patterns congruent with those of a phyllosphere microbiome that mediates host physiology” this argument goes both ways - and can easily be explained by the plant host shaping the microbiome and not the microbiome shaping the host.

**Author response:** We thank the reviewer for raising this point and have reworded the corresponding text. The text now reads as follows:

Lines 21-26:

*“Working within the prevailing core microbiome conceptual framework, we hypothesise that the phyllosphere microbiome of mānuka exhibits specific host association patterns*

*congruent with those of a microbial community under host selective pressure (null hypothesis: the mānuka phyllosphere microbiome is recruited stochastically from the surrounding environment).”*

8. Line 52-64 neither me nor the other reviewer asked for this - and it is not appropriate in the context of a research paper - simply write which definition you use like you do any in line 46 - here you already write "the leaf surface, or phyllosphere" so this whole discussion is distracting from the study. I also cannot follow the whole point of this paragraph - and suggest to delete to whole paragraph from line 52-64.

additionally reference 10 does not support your statement. Whipps does not include the endophytic compartment in the phyllosphere - if you read carefully, they are writing about a surface area and distinguish phyllosphere bacteria from endophytes: Phyllosphere bacteria can promote plant growth and both suppress and stimulate the colonization and infection of tissues by plant pathogens (Lindow and Brandl 2003; Rasche et al. 2006a). Similarly, fungal endophytes of leaves may deter herbivores, protect against pathogens and increase drought tolerance (Arnold et al. 2003; Schweitzer et al. 2006).

**Author response:** As stated by the reviewer in comment #10 of the previous review, the phyllosphere is inconsistently defined across scientific literature. We therefore felt it was important to clarify our definition in our manuscript and by doing so provide important background information to the forthcoming notion that the concept of core microbiome is in fact not well studied for the phyllosphere. However, we agree that including such a paragraph is slightly distracting from the study and therefore, as requested by the reviewer, we have deleted this paragraph from the introduction.

9. Regarding reference 11 this review is really not great - first off, I agree that they falsely define the phyllosphere by including the endophytic compartment, they are very alone in this. They also proclaim highly problematic messages which were impressively dismissed just a year later it starts with "Most microorganisms of the phyllosphere are nonculturable in commonly used media and culture conditions" e.g. Bai et al. 2015 (nature) and many others showed that indeed most bacteria from leaf surfaces can be cultivated.

**Author response:** In response to comment #8 where the reviewer requested the removal of the paragraph from lines 52-64, this citation has been removed.

10. Line 77 references 9,24 and 28 are not an appropriate reference here - these studies do not investigate interactions of the microbiota with the plant host instead the are investigating community structure but not micro-host interactions.

**Author response:** We thank the reviewer for pointing out this mistake. We have removed references 9 and 24 as requested and have included citations that directly demonstrate interactions between microbiota and the plant host. Kembel et al. (2014) (original reference #28) provide evidence for evolutionary associations between plants and phyllosphere bacteria. We have therefore kept this citation as we feel this study provides important information that is relevant to the text. The text now reads as follows:

*Line 76-78: “However, the existing evidence does indicate that phyllosphere microbiomes exhibiting host-specific associations are more likely to interact with the host than those primarily recruited from the surrounding environment [5, 17-19].”*

*[5] Vogel C, Bodenhausen N, Gruissem W, Vorholt JA. The Arabidopsis leaf transcriptome reveals distinct but also overlapping responses to colonization by*

*phyllosphere commensals and pathogen infection with impact on plant health. New Phytologist. 2016;212(1):192-207.*

[17] *Kembel SW, O’Connor TK, Arnold HK, Hubbell SP, Wright SJ, Green JL. Relationships between phyllosphere bacterial communities and plant functional traits in a neotropical forest. Proceedings of the National Academy of Sciences. 2014;111(38):13715-20.*
[18] *Innerebner G, Knief C, Vorholt JA. Protection of Arabidopsis thaliana against leaf-pathogenic Pseudomonas syringae by Sphingomonas strains in a controlled model system. Appl Environ Microbiol. 2011;77(10):3202-10. Epub 2011/03/23. doi: 10.1128/aem.00133-11. PubMed PMID: 21421777; PubMed Central PMCID: PMCPMC3126462.*
[19] *Lajoie G, Maglione R, Kembel SW. Adaptive matching between phyllosphere bacteria and their tree hosts in a neotropical forest. Microbiome. 2020;8(1):70. doi: 10.1186/s40168-020-00844-7.*
11. Line 128 - 130 this sentence still suggests that you would be investigating DHA variations - I only agree with the second part of the sentence that the mānuka phyllosphere microbiome is underexplored. Reword the sentence to avoid overstressing DHA. Instead the authors may claim that the knowledge that they build here can in the future be useful to explore reasons for differing DHA concentrations.

**Author response:** We thank the reviewer for their advice to reword this sentence and agree with the reviewer’s comment that the knowledge built here may be useful in the future to explore reasons for differing DHA concentrations. To avoid overemphasising our findings’ relevance for DHA, we have reworded the sentence as suggested. This now reads as follows:

Lines 108-115:

*“Microorganisms have been studied in the mānuka rhizosphere and endosphere [40- 42]. Although existing studies have primarily focussed on fungi, a recent study provided the first investigation of endophytic bacterial communities from three geographically and environmentally distinct mānuka populations using fingerprinting techniques and revealed tissue-specific core endomicrobiomes [43]. However, a similar characterisation of the mānuka phyllosphere microbiome has not been conducted.”*

12. Line 131 - 134 and again the authors are stressing that they will deliver answers to why DHA concentrations differ - your study does not contribute to extending the knowledge of this particular aspect and can only function as an hypothesis generator

**Author response:** It was not our intention to imply that this study delivers answers regarding DHA heterogeneity, and we apologise for giving the reviewer this incorrect impression. We agree with the reviewer’s comment that this study functions as a hypothesis generator to explore reasons for differing DHA concentrations in the future. We have made changes to the text to clarify this:

Lines 116-127:

*“Inspired by the intriguing physiological characteristics of the mānuka tree, and the increasingly recognised role of phyllosphere bacteria in plant physiology, we provide the first characterisation of the bacterial communities comprising the mānuka phyllosphere microbiome. Working within the prevailing core microbiome conceptual framework, we hypothesise that phyllosphere microorganisms of mānuka exhibit*

*specific host association patterns congruent with those of a microbial community under host selective pressure. Correspondingly, our null hypothesis is that the mānuka phyllosphere microbiome is recruited stochastically from the surrounding environment.”*

13. General comment- there is a dichotomy between the core microbiome concept and the heterogeneity in DHA - which is still a prevailing problem of the underlying hypothesis: if the core microbiome were to be responsible for DHA formation, it should be more uniform. Instead, the accessory microbiome seems to have larger effects on the formation of DHA. Despite the claims of the authors that they adjusted their writing this is still reads like this is connected.

**Author response:** At present, the cause of DHA heterogeneity in mānuka nectar remains a scientific enigma. We would like to reassure the reviewer that it was not our intention to imply that the core microbiome (if present) is responsible for DHA formation. Please see specific comments #11, #12, and #14 where we have made specific changes regarding DHA.

A core microbiome at the OTU level does not imply strain-level homogeneity in composition nor function. In other words, a core microbiome at the OTU level may exhibit large functional diversity across individual hosts and thus effect divergent host physiological responses. For example, Karasov et al. (2018) (doi:10.1016/j.chom.2018.06.011) performed an investigation of leaf-associated *Pseudomonas* populations on wild *Arabidopsis* and showed that the OTU that was found most commonly on (and in) leaves (called OTU5, based on 99% identity at 16S rRNA gene level) represented 165 different strains or genomes.

Many core microbiomes reported in the scientific literature have been predominantly defined using genus-level discrimination of a population (i.e., Rastogi et al. 2012, doi: 10.1038/ismej.2012.32), and strain-specific as well as functional variation are often not immediately distinguished. Identification of a core microbiome at the OTU level and the potential for extensive strain-level diversity offers an exciting point-of-departure for future inquiries into the functional role of phyllosphere-associated microbial taxa and communities.

14. Line 148-150 Both reviewers asked you to remove this statement and this reviewer stands to this statement. Remove the claims that this increases the understanding about if microorganisms change DHA concentrations if DHA and or host physiology were not investigated.

**Author response:** It was not our intention to imply that this study increases the understanding about whether microorganisms change DHA concentrations. Rather, as acknowledged by the reviewer in comment #11, the knowledge built here may be useful in the future to explore reasons for differing DHA concentrations. We apologise for our lack of clarity in the text and have revised this statement as per the recommendations of the reviewer:

*Lines 130-137:*

*“Our findings revealed a dominant and ubiquitous core microbiome, providing strong evidence for a specific host association. This knowledge may be useful for designing future studies that will enhance our understanding of plant-microbe associations in the mānuka phyllosphere and address significant and longstanding questions such as the factors driving spatiotemporal variability in mānuka DHA production.”*

15. Line 457-465 I reiterate - the PiCrust analysis does not add to the study and it is not discussed at all. It should be deleted. It also does not add worthwhile intel but rather highlights that in the future, metagenomic and transcriptomic studies should be conducted. Delete it

**Author response:** As requested by the reviewer, the PICRUSt analysis has been removed from the manuscript and supplementary material.

16. Line 505 regrading Terriglobus - now that is exciting - what is the known about this organism?

**Author response:** We thank the reviewer for raising this question. *Terriglobus* is a member of the phylum *Acidobacteria*, which are widely distributed and abundant in soils. Physiological characteristics of *Terriglobus* are consistent with an oligotrophic lifestyle, which is particularly interesting as the phyllosphere is also considered an oligotrophic environment (Eichorst et al, 2007, doi:10.1128/AEM.02140-06). We have included further information regarding Terriglobus in the text.

Lines 525-527:

“*Other core OTUs were assigned to genera not commonly reported in the phyllosphere, such as Terriglobus, which are generally considered well adapted to survive oligotrophic environments [72].”*

17. Line 518 - 535 With your study you show that a limited number of species are part of a core microbiome, you see this as evidence that they are important for community functioning and plant health but based on this data it is not possible if they are “key- stone” taxa (for further explanation about keystone taxa in the phyllosphere check e.g. the following

papers https://journals.plos.org/plosbiology/article?id=10.1371/journal.pbio.1002352 [http](https://pubmed.ncbi.nlm.nih.gov/31558832/) [s://pubmed.ncbi.nlm.nih.gov/31558832/](https://pubmed.ncbi.nlm.nih.gov/31558832/)). Since such an analysis is not possible and the results provided to not support the claims the authors make, I reject the response of the authors to point 4 of my previous review. No evidence was provide to support their claims.

**Author response:** We agree with the reviewer that it is not possible to directly infer whether the core microbiome identified here are “keystone” taxa, based on the analyses presented in this study. The tenet of keystone taxa was originally proposed by ecologist Robert T. Paine in 1966. In a classic experiment, he demonstrated that removing sea stars (*Pisaster ochraceus*) had a significant impact on the shoreline ecosystem community and biodiversity at Makah Bay, Washington, USA. The classic experimental validation of keystones involves comparing the effects of keystone removal and/or addition on other community members. As experiments such as these are difficult to carry out on microbial communities, only few microbial taxa have actually been experimentally confirmed as keystones (Garrett et al. 2010, doi:10.1016/j.chom.2010.08.004; Hajishengallis et al. 2011, doi:10.1016/j.chom.2011.10.006; Ze et al. 2012, doi:10.1038/ismej.2012.4; Agler et al. 2016, doi: 10.1371/journal.pbio.1002352).

Studying the community structure and composition of a host microbiome provides valuable information that can be used to generate hypotheses regarding genetic, chemical, or microbial targets for future studies, such as Agler et al. 2016 (doi:10.1371/journal.pbio.1002352) and Carlstrom et al. 2020 (doi:10.1038/s41559-019- 0994-z). The overall community structure (i.e., a prominent/dominant core microbiome across broad geographical distances) is consistent with the notion that the mānuka

phyllosphere microbiome exhibits specific host association. We do not intend to claim anything more than these bacteria *may* be important/worthwhile looking at into in the future.

18. Line 530 diazotrophic nitrogen fixation is unlikely to be a major function on leaves in temperate conditions since it requires absence of oxygen reference 51 refers to tropical rain forest leaves which cannot be compared to temperate leaves since the leaves develop stratified biofilms that allow for anoxic niches. Reference 70 Delmotte et al did not investigate if the bacteria actually “provide various nutrients, phytohormones, and precursors for essential plant metabolites”. What we do know is that methylobacteria species and others metabolise methanol, some bacteria produce phytohormones (check studies regarding IAA of Leveau and others, or Rhodococcus that produce cytokine in studies from Danny Vereecke or Paula Jameson)

**Author response:** We thank the reviewer for making this point and offering these citations. As requested by the reviewer, we have removed reference 51 (Furnkranz et al. 2008) and 70 (Delmotte et al. 2009) from line 530 and revised the sentence.

Lines 546-548:

*“Rhizobiales also contains many genera of methylotrophic bacteria that that metabolise methanol and enhance plant growth by producing phytohormones, such as indole-3-acetic acid [75, 76].”*

[75] *Ashok G, Nambirajan G, Baskaran K, Viswanathan C, Alexander X. Phylogenetic Diversity of Epiphytic Pink-Pigmented Methylotrophic Bacteria and Role in Alleviation of Abiotic Stress in Plants. In: Yadav AN, Singh J, Rastegari AA, Yadav N, editors. Plant Microbiomes for Sustainable Agriculture. Cham: Springer International Publishing; 2020. p. 245-62.*
[76] *Meena KK, Kumar M, Kalyuzhnaya MG, Yandigeri MS, Singh DP, Saxena AK, et al. Epiphytic pink-pigmented methylotrophic bacteria enhance germination and seedling growth of wheat (Triticum aestivum) by producing phytohormone. Antonie van Leeuwenhoek. 2012;101(4):777-86. doi: 10.1007/s10482-011-9692-9.*
19. The discussion ends on a discussion around contamination this give a lot of emphasis on the used methodology however, previously asked to remove this for a reason and I will support this here once more: the claims that the authors make a merely based on assumptions and were not benchmarked against other established techniques. Instead I would move parts of this paragraph into the material and methods and indicate which educated decisions were made to change existing protocols.

**Author response:** We thank the reviewer for confirming this point raised in the first review and for considering our rationale for keeping this discussion regarding contamination. As requested by the reviewer, parts of this paragraph have now been removed from the Discussion and incorporated into Material and Methods.

The Materials and Methods now reads as follows: Lines 173-181:

## “Bacterial community collection

*Per branch, 1 g of predominantly healthy, undamaged green leaves were aseptically excised and pooled. Leaves were submerged in 10 mL of wash buffer (phosphate buffer solution [PBS, 100mM NaH2PO4], 1% tween 20) and sonicated for 20 min (60 Hz) in an ultrasonic cleaning bath. Sonication was used to remove bacteria from the leaf surface in order to minimise undesirable amplification of chloroplast rRNA genes*

*and contamination by endophytic bacteria, which are typically recovered via methods that inflict damage to leaves such as maceration and coring [44, 45]. After sonication, the wash buffer was decanted, syringe filtered through 90 µm nylon mesh to remove finer plant debris, and centrifuged (3,200 x g for 30 minutes).”*

Lines 194-207

## “DNA library preparation and sequencing

*Total community DNA extracted from the phyllosphere and surface soil was used for amplification and sequencing of the V4 region of the 16 rRNA gene using the universal primer set F515 (5’GTGCCAGCMGCCGCGGTAA-3’) and R926 (5’- CCACTACGCCTCCGCTTTCCTCTCTATGGGCAGTCGGTGATCCGYCAATTYMTT*

*TRAGTTT-3’). Sample cross-contamination via aerosolised PCR products is a major limitation of two-step protocols widely used in preparing 16S rRNA gene PCR amplicons for Illumina MiSeq sequencing [47-51]. To avoid cross-contamination of aerosolised PCR products, we used fusion primers to prepare 16S rRNA gene PCR amplicons in a one-step protocol. Unlike previous studies, chloroplast-excluding primers were avoided in order to prevent taxonomic bias and permit identification of Cyanobacteria in the mānuka phyllosphere [52-54]. The fusion primers used in the current study generated an amplicon of approximately 500 bps with adapters suited for Ion Torrent sequencing.*

The Discussion now reads as follows: Lines 599-633:

## “Future directions

*Our study was strengthened by aspects of our methodological design that allowed us to essentially exclude cross-contamination as an alternative explanation for the presence of a ubiquitous core microbiome. For example, soil was processed simultaneously with leaf samples to address potential concerns of core taxa being an artefact of contamination during sample collection or processing, and fusion primers were used to prepare 16S rRNA gene PCR amplicons in a one-step protocol mitigate the risk of aerosolised PCR products [47-51]. The immediate next step should be to investigate the functional potential and role of the core phyllosphere microbiome in mediating the physiological properties of mānuka using a combination of shotgun metagenomic sequencing and cultivation approaches. Metagenomics will also reveal the diversity and abundance of micro-eukaryotes such as fungi and protists, which are increasingly recognised as potential determinants of plant-microbe interactions and host fitness. Incorporating host genetic analyses and co-occurring plant species into future analyses will also shed light on the relationship between the mānuka phyllosphere microbiome and host genetics as well as the role of recruitment in community assembly.”*

The Conclusion now reads as follows: Lines 634-664:

## “Conclusion

*Our findings demonstrate that Leptospermum scoparium (mānuka) possesses a dominant core phyllosphere microbiome that is persistent across geographically distant populations. Core phyllosphere taxa represented a large proportion of the total phyllosphere community yet were rare or entirely absent in surrounding soils, providing support for hypothesised specific host association and potentiating the role of host selection of functionally important microorganisms. Strong biogeographical patterns were also identified in the mānuka phyllosphere, indicating that spatially structured environmental gradients may play a role in shaping the composition of non-core*

*microbiota. Together, these findings illustrate that the mānuka phyllosphere microbiome exhibits complex biogeography that likely resulted from divergent processes driving the assembly of core and non-core microorganisms. Understanding potential roles of the mānuka phyllosphere microbiome in the physiology of the nationally and culturally treasured indigenous plant will be of wide scientific and general interest. Our findings also illustrate the usefulness of the mānuka phyllosphere microbiome for expanding our knowledge of specific host-microbe associations in the phyllosphere and support the notion that investigation of phyllosphere microbiomes should be conducted as per our sensu stricto definitions of the phyllosphere and core microbiome to ensure relevance and generalisability of findings.”*
